# Surgical Management of Acute Type A Aortic Dissection

**DOI:** 10.31083/RCM54094

**Published:** 2026-10-13

**Authors:** Adam H Kiridly, Michael A Catalano, Branden Tejada, Michele Dell’Aquila, Isabella Kola, Omar A Jarral, Stevan S Pupovac, Derek R Brinster

**Affiliations:** ^1^Department of Cardiothoracic Surgery, Northwell Cardiovascular Institute, Manhasset, NY 11030, USA; ^2^Division of Cardiac Surgery, Hospital of the University of Pennsylvania, Philadelphia, PA 19104, USA

**Keywords:** acute aortic syndrome, aortic dissection, ascending aorta, thoracic aorta

## Abstract

Acute type A aortic dissection (ATAAD) is a life-threatening cardiovascular emergency characterized by disruption of the ascending aortic intima with subsequent propagation of a false lumen. Population-based studies report an annual incidence of approximately 3 cases per 100,000 patient-years. Despite advances in diagnosis and surgical management, 30-day operative mortality remains approximately 10–20%, with higher rates in the presence of malperfusion syndrome, cardiac tamponade, or aortic rupture. This narrative review summarizes the current understanding of ATAAD pathophysiology, classification systems, diagnostic modalities, and evolving management strategies, with particular emphasis on contemporary surgical techniques and organ protection strategies employed during repair. Hypertension remains the most significant modifiable risk factor for ATAAD, whereas hereditary thoracic aortic disease (HTAD) predisposes younger patients to dissection. The Penn classification stratifies mortality risk according to the pattern of malperfusion; coronary and mesenteric malperfusion are associated with the highest mortality. Surgical management has evolved from traditional hemiarch replacement to more extensive arch replacement, with or without frozen elephant trunk (FET) techniques, and emerging data demonstrate improved long-term durability. Moderate hypothermic circulatory arrest (MHCA) with antegrade cerebral perfusion (ACP) has emerged as the preferred neuroprotective strategy, offering outcomes comparable to those achieved with deep hypothermic circulatory arrest (DHCA) alone. Treatment at high-volume comprehensive aortic centers is associated with improved survival. Management of ATAAD continues to evolve through ongoing refinements in surgical techniques, organ-protection strategies, and perioperative care. While prompt surgical intervention remains the cornerstone of treatment, individualized management that incorporates patient-specific anatomy, malperfusion patterns, and institutional expertise is essential. Long-term surveillance and follow-up at specialized aortic centers are critical for detecting and managing late complications, and genetic evaluation is recommended to identify HTAD in appropriate patients.

## 1. Introduction

Acute aortic syndrome (AAS) refers to a group of potentially fatal disruptions of the aortic intima, including intramural hematoma, penetrating aortic ulcer, and aortic dissection. In the setting of aortic dissection, an intimal tear allows blood to enter the media, forming a false lumen that propagates along the vessel [1,2,3]. Acute type A aortic dissection (ATAAD) describes any aortic dissection that involves the ascending aorta [4]. Emergency surgical evaluation is recommended due to the high risk of progression to aortic rupture, cardiac tamponade, acute aortic regurgitation (AR), and malperfusion syndrome [4,5,6,7]. The management of ATAAD has evolved substantially over recent decades, driven by advances in imaging and surgical techniques, including open and hybrid repairs [4,5,6].

## 2. Methods

Given the significant changes in our understanding of ATAAD in the last decade, the goal of this narrative review is to contextualize these new data, and explore emerging management strategies. References were selected for inclusion based on a query of Embase, PubMed, and Medline/OVID databases to identify studies published since 2010 using relevant keywords including “acute type A aortic dissection”, “acute aortic syndrome”, etc. Previous society-generated guidelines for management of ATAAD were reviewed, and an iterative process was used to add additional references to contextualize and evaluate these new publications [1,4,7,8,9,10].

## 3. Incidence and Severity

Meta-analysis of the incidence of ATAAD in population-based studies reports a mean annual incidence of about 3 per 100,000 patient-years [11]. However, given the rate of pre-hospital mortality for ATAAD, its true prevalence is likely higher. Post-mortem computed tomography was used to identify a 7% incidence of ATAAD in patients with pre-hospital cardiopulmonary arrest, and reported a 51% pre-hospital mortality rate for ATAAD [12]. Non-operative ATAAD management is associated with 57% mortality, while 30-day operative mortality for ATAAD ranges from 10–20% based on contemporary multi-center studies and registry reviews, even higher (27% to 42%) in patients with associated malperfusion syndrome, pericardial tamponade, and aortic rupture [4,5,6,9,13].

## 4. Etiology and Risk Factors

Proposed mechanisms of ATAAD include a combination of underlying pathology that predisposes the aorta to mechanical failure, and a stimulating stress event that overwhelms aortic compliance leading to intimal tear formation [14,15]. Once an intimal tear develops, pressurized blood dissects into the media, forming a false lumen, separated from the true lumen by an intimal–medial flap. The release of chemokines from disrupted aortic smooth muscle cells leads to further inflammation, and weakening of the aortic wall [16].

Hypertension is the most significant modifiable risk factor for ATAAD development with an attributable risk of 54%, with tobacco smoking and dyslipidemia carrying attributable risks of 14% and 30%, respectively [17]. Notably, hereditary thoracic aortic disease (HTAD) that results in compromised integrity of the aortic media, including Marfan (0.8–12% prevalence of ATAAD), vascular Ehlers-Danlos (1.3–5.8%), Loeys–Dietz (16%), and Turner (1–5%) syndromes, is associated with early presentation of aortopathies [4,5,15,18]. Presence of bicuspid aortic valve, male sex, and advanced age are also associated with increased risk of ATAAD [5,6]. Pregnancy is a known trigger for ATAAD due to hemodynamic and hormonal changes that stress the aortic wall [19]. Before planned pregnancy, patients with HTAD are recommended to meet with a multidisciplinary team, undergo genetic counseling, and undergo surveillance aortic imaging [20,21,22]. In pregnant patients with HTAD, guideline-directed hypertension treatment can help prevent aortic dissection.

## 5. DeBakey, Stanford, TEM, and SVS/STS Classifications

The DeBakey classification categorizes aortic dissections based on the origin and longitudinal extent of the intimal tear [23]. The Stanford classification (Fig. 1A) is a widely adopted alternative that sorts aortic dissection into two groups based on the involvement of the ascending aorta. Both historic systems have limitations, especially in describing aortic dissections that begin in the aortic arch or involve complex propagation patterns. The Type-Entry-Malperfusion (TEM) classification (Fig. 1B) characterizes the dissection’s extent, the entry location, and the presence of malperfusion [24]. These classification systems are described in Table 1.

**Fig. 1.**
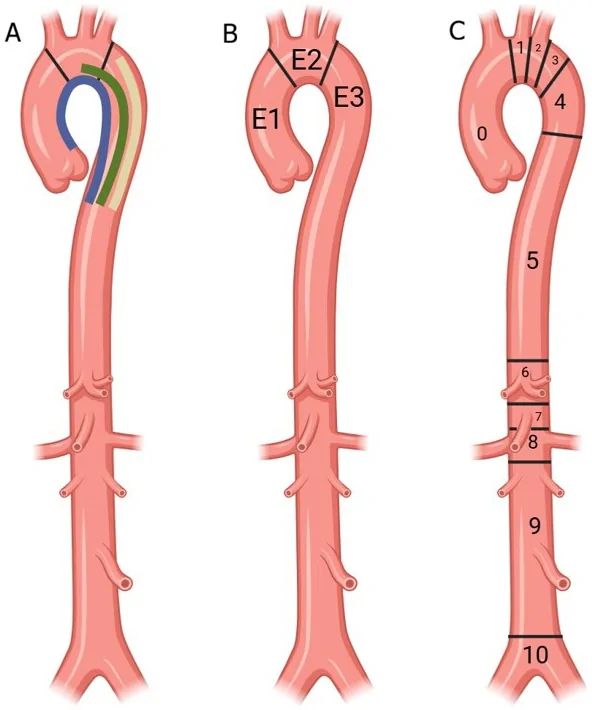
** Aortic dissection classifications**. (A) Blue line: Stanford type A dissection; Beige line: Stanford type B dissection; Green line: non-A, non-B dissection. (B) TEM Entry locations. (C) STS/SVS zones. TEM, Type-Entry-Malperfusion; SVS/STS, Society for Vascular Surgery/Society of Thoracic Surgeons. Created in BioRender.

**Table 1.** **DeBakey, Stanford, and TEM classification systems**.

DeBakey	**Type I:** originates in the ascending aorta and propagates through the arch and distally **Type II:** originates in, and is confined to, the ascending aorta **Type III:** originates in the descending aorta and rarely extends proximally
Stanford	**Type A:** dissection involving the ascending aorta, regardless of where the tear originates **Type B:** dissection doesn’t involve the ascending aorta. Entry tear distal to the left subclavian **Type non-A non-B:** dissection involves the arch and descending but not the ascending
TEM	**Type: ** Stanford A, B, or non-A non-B **Entry: ** E0: no identifiable entry E1: entry in the ascending between the aortic valve and the proximal edge of the innominate E2: entry in the arch between the innominate and the distal edge of the left subclavian E3: entry in the descending aorta **Malperfusion:** M0: no clinical or radiologic malperfusion M1: dissection of at least 1 main coronary artery with or without myocardial malperfusion M2: dissection of at least 1 supra-aortic vessel or aortic arch true lumen collapse with or without cerebral or upper limb malperfusion M3: dissection of at least 1 visceral, renal or iliac artery or aortic true lumen collapse with or without mesenteric, renal, or lower limb malperfusion

The SVS and STS proposed a more granular classification (Fig. 1C) based on Ishimaru zones of proximal and distal extent of dissection as well as the location of the primary entry tear [7,20,25]. In this system, the aorta is broken up into zones 0–12 from the ascending aorta to the iliac arteries. The classical anatomic classifications remain essential for rapid clinical triage and surgical decision-making, while the newer systems enhance precision in describing disease patterns that may influence management and outcomes, especially as endovascular management options of acute dissection are pursued. TEM is useful for prognostication and outcomes prediction, while STS/SVS provides detail for operative planning, with Stanford and DeBakey offering the fastest ways to communicate type of dissection.

## 6. Acuity

The timing of symptom onset in ATAAD has important implications for prognosis and management, with timing typically stratified as hyperacute, acute, subacute, or chronic [6,25]. Traditionally, dissections were dichotomized simply as acute (<14 days) versus chronic (≥14 days) from symptom onset to clinical presentation [23]. Investigators of the International Registry of Acute Aortic Dissection (IRAD) data further refined this approach by identifying four distinct inflection points in survival curves, regardless of management type, for ATAAD: hyperacute (symptom onset to presentation <24 hours), acute (2–7 days), subacute (8–30 days), and chronic (>30 days) [26]. These findings emphasize the importance of prompt identification and management of ATAAD [6,27,28].

## 7. Clinical Presentation and Diagnosis

Patients with ATAAD typically present with abrupt onset of severe pain, most commonly in the chest or upper back and often described as tearing, ripping, or sharp, with maximal intensity at onset. On physical examination, patients may exhibit widened pulse pressure, new diastolic murmur (representative of AR), asymmetrical or diminished peripheral pulses, and interarm blood pressure discrepancies. Muffled heart sounds, distended jugular veins, hypotension, and electrical alternans on electrocardiogram (ECG) may indicate aortic rupture into the pericardium [4,5,6,29]. End-organ malperfusion can result in varied presentation (see Malperfusion section).

When clinical suspicion for ATAAD is high, computed tomography (CT) angiography (CTA) of the chest, abdomen, and pelvis should be pursued promptly to confirm the diagnosis and identify the location of the intimal tear, the extent of the dissection, patterns of flow in the true and false lumens, branch-vessel involvement, and any associated pericardial or pleural effusions [29,30,31]. For stable patients, adjunctive tools such as echocardiography and biomarkers (e.g., lactate, troponins, and creatinine) may assess for malperfusion. In hemodynamically unstable patients who may not tolerate CTA, preparation for emergency surgical intervention should occur simultaneously with emergent imaging modalities such as bedside transthoracic echocardiography and transesophageal echocardiography (TEE) [29,31].

Once imaging confirms the diagnosis, prompt activation of the surgical team is essential [4,9]. Future directions of study include improving risk-prediction models to identify high-risk individuals for perioperative complications based on imaging findings, biochemical markers, and patient factors, beyond those that exist currently (see Malperfusion syndrome section).

## 8. Initial Management and Stabilization

Initial management of ATAAD focuses on immediate medical stabilization to reduce aortic wall shear stress, limit propagation of the dissection, control pain-induced sympathetic activation, and preserve end-organ perfusion while preparing for definitive surgical repair [3,4,31,32,33]. Patients should undergo continuous telemetry and blood pressure monitoring, with prompt establishment of large-bore intravenous access [34]. Monitoring and documentation of peripheral pulses and neurologic exam are essential for early identification of malperfusion symptoms both in the preoperative and postoperative setting. Placement of arterial lines in both the upper and lower body may be helpful, particularly in patients with concern for arch vessel or lower extremity malperfusion [7,35]. This allows for monitoring of peripheral perfusion during and after intervention [34]. However, placement of these lines should not delay definitive operative management. Adequate analgesia, typically with intravenous opioids, should be administered early to relieve pain and blunt the catecholamine surge that can exacerbate hypertension and shear stress [1].

The cornerstone of medical therapy for ATAAD is anti-impulse therapy, with intravenous β-blockers as first-line agents, targeting a heart rate of less than 60 beats per minute and systolic blood pressure of 100–120 mmHg [9,29]. Esmolol is favored for its rapid onset and short half-life, but labetalol can be used as an alternative [9,32,36]. For patients with cardiac tamponade, emergent surgery should be performed, with consideration of the use of controlled pericardiocentesis only in the case of tamponade-related hemodynamic instability, as a bridge therapy in the case of substantial delay from presentation to appropriate definitive surgical management [1,37,38].

## 9. Timing of Intervention

Given the high risk of aortic rupture, cardiac tamponade, and death associated with ATAAD, current guidelines recommend immediate surgical evaluation and emergent operative repair once the diagnosis is confirmed or strongly suspected in high-risk patients [4,9]. Older studies have consistently demonstrated a clear time-dependent risk of mortality with delays to definitive repair, with mortality increasing by approximately 1–2% per hour in the first day in the absence of surgery, with a recent report describing a 2.6% hourly mortality rate for the first day [6,9,26,27,28,39,40].

## 10. Transfer Considerations to Dedicated Aortic Surgical Centers

Prior studies have demonstrated that patients with ATAAD experience reduced risk of morbidity and mortality when undergoing surgery at high-volume aortic institutions [41]. When ATAAD is confirmed at a non-surgical facility, rapid transfer to a dedicated aortic surgery center with ongoing medical stabilization is recommended for hemodynamically stable patients [42,43,44]. In unstable patients, emergent local surgery is appropriate if immediately available; otherwise, carefully selected patients may still experience significantly improved survival outcomes after expedited transfer to an aortic surgery center following limited stabilization, coordinated closely with the receiving center [42,43,44,45,46,47]. Efficient interfacility transfer, with direct clinician communication and prompt transmission of imaging and clinical data, reduces delays. In fact, patients transferred alive for definitive repair may achieve outcomes comparable to direct admissions when transfer is timely and well-coordinated [48]. While time from admission to surgery at a dedicated aortic surgery center for ATAAD has significantly decreased from a median of 7.1 hours to 6.5 hours, there continues to be room for improvement with education and quality control efforts at centers of all levels of care [49].

Given the emergent nature of ATAAD management, organizations have implemented dedicated aortic emergency phone numbers for referring hospitals, direct-to-operating room aortic emergency transfer programs, and algorithms designed to streamline steps between the presenting emergency department and operating room of the definitive aortic center [50]. When the external site refers a patient to the aortic surgical team, the decision to accept transfer to the OR immediately activates the OR team such that, when the patient arrives, the site of definitive management is ready for immediate management [50]. This results in significantly decreased time from transfer acceptance to OR and time from hospital arrival to OR [50]. Organizational development of aortic surgery teams is critical in providing timely care for patients with aortic pathologies [42,51].

## 11. Malperfusion Syndrome

Pre-operative malperfusion in patients with ATAAD occurs with an incidence of 15–40% and is an independent risk-factor for in-hospital mortality [6,9,13,52]. In patients with ATAAD, malperfusion syndrome is associated with a significantly higher rate of in-hospital mortality than patients without malperfusion (30.5% vs 6.2%) [53]. Malperfusion, or inadequate blood supply to an end organ, can lead to malperfusion syndrome, which results from sustained ischemia of one or more end-organs, often as a result of branch vessel obstruction.

Physiologic presentations of malperfusion include neurologic, cardiac, renal, mesenteric, and limb. Cerebral malperfusion may present with syncope and neurological deficits, including coma and stroke [5,6,29]. Spinal malperfusion may present with motor or sensory deficits in the lower extremities [9]. Coronary malperfusion may present with chest pain and cardiogenic shock in the setting of myocardial ischemia or infarction [9]. Renal malperfusion may manifest as decreased urinary output. Mesenteric malperfusion presents with abdominal pain, and limb ischemia may present with limb pain, and motor and/or sensory deficits [13,53,54,55,56,57].

Preoperative malperfusion represents an important predictor of early mortality after surgical repair [13,53,58,59,60,61]. The University of Pennsylvania (Penn) classification system is a tool used to understand the impact of malperfusion on surgical outcomes. Patients are classified into one of four classifications (A, B, C, and B-C). A represents hemodynamic stability without signs or symptoms of ischemia. B represents hemodynamic stability with evidence of branch vessel malperfusion or symptoms of localized ischemia (e.g., stroke, ST segment changes, abdominal pain, limb ischemia). C represents global malperfusion from circulatory collapse. B-C involves global malperfusion from circulatory collapse plus evidence of local ischemia [52]. In a recent single-center review validating the Penn classification, Patrick and colleagues [52] identified the following 30-day mortality risk, stratified by Penn classification: A–5.2%, B–10.3%, C–15.2%, B-C–35.3%. There are several risk stratification tools validated for predicting mortality risk in patients with ATAAD with or without malperfusion that perform reasonably well including Penn (area under the curve (AUC) 0.77), German Registry for Acute Aortic Dissection Type A (GERAADA) (0.73), and ALICE (0.83) [1,5,13,27,52,59,62].

In addition to classification systems that stratify patients by degree of malperfusion, such as the Penn classification, specific malperfusion location is highly associated with mortality risk. A recent review of the STS database identified the following rates of malperfusion in a contemporary series of surgically repaired ATAAD from 2017–2020: cerebral (9.8%), coronary (5.0%), renal (10.2%), mesenteric (5.7%), extremity (14.9%), and spinal malperfusion (1.0%) [13]. In this study, the authors identify the highest adjusted odds of 30-day mortality associated with coronary (OR 2.36) and mesenteric (OR 1.84) malperfusion, which is consistent with other large series [9,13,53,54,55,60].

Traditionally, immediate proximal aortic repair has served as the primary management of pre-operative malperfusion, restoring true lumen flow and resolving branch-vessel occlusion [9,53]. Surgical intervention mitigates the immediate risk of fatal complications, corrects AR and myocardial ischemia, and often reestablishes flow to malperfused vessels [9,53]. In some cases, peripheral revascularization may be performed in the setting of ongoing malperfusion following aortic repair.

More recently, select centers have advocated for targeted revascularization of mesenteric malperfusion in stable ATAAD patients using endovascular fenestration, branch-vessel reperfusion, or hybrid before primary open repair [63,64]. By restoring mesenteric perfusion via endovascular approaches before proximal aortic repair, the risk of ongoing mesenteric ischemia during cardiopulmonary bypass (CPB) is minimized. Various centers have reported reduced risk of in-hospital mortality associated with this approach [9,63,64,65]. However, it is important to note that delayed proximal aortic repair is not without risk, and patients may experience increased risk of aortic rupture during complex mesenteric reperfusion procedures [9,63]. For example, Yang and colleagues [64] describe their approach to endovascular intervention on mesenteric malperfusion with delayed proximal aortic repair; in the first decade of their series, 16% died from aortic rupture before open repair, improving to 4% in their second decade. However, those who reached open surgical intervention for proximal repair experienced a remarkably low operative mortality of 2% [64]. Additionally, Leshnower and colleagues [63] performed a single-institution retrospective review of a thoracic endovascular aortic repair (TEVAR)-first approach to ATAAD with mesenteric malperfusion. Patients with mesenteric malperfusion without shock, tamponade, rupture, or severe AR were selected to undergo TEVAR-first; while this likely represents a lower risk cohort, the TEVAR-first patients experienced a significantly reduced in-hospital mortality of 30%, versus 69.2% in the cohort of patients undergoing primary open repair first [63]. TEVAR-first management of mesenteric malperfusion is an investigational strategy, with expert aortic care centers demonstrating reasonable outcomes, while current guidelines support proximal aortic surgery first [63,64,65,66]. Future multicenter prospective investigations into TEVAR-first management will help inform future guideline development.

The case of ATAAD complicated by cerebral malperfusion is associated with a significant increase in morbidity and mortality [52,55,60,67]. In the case of cerebral malperfusion with carotid occlusion, no evidence-based consensus has been established [67,68,69]. Surgical options include restoration of cerebral blood flow via conventional central aortic repair or early restoration of cerebral perfusion (either via early operative branch perfusion, stenting, or via transcatheter carotid artery perfusion cannula placement) [68]. While central aortic repair continues to be the most common practice, in cases of occlusion of the common carotid arteries, cannulation or anastomosis distal to the carotid occlusion to establish early antegrade cerebral perfusion (ACP) has been performed to minimize cerebral ischemia time before central repair. This is often followed by carotid transection and false lumen thrombectomy then aortic reconstruction [68]. Others have found preliminary success using MRI to evaluate for salvageable cerebral tissue, and if salvageable, performing early carotid artery stenting [69].

In addition to tailoring repair approach based on presence and type of malperfusion, other centers have advocated for stratifying patients based on timing of presentation. A recent, single-center retrospective review assessed the outcomes of a traditional approach to an individualized approach with situational reperfusion-first ATAAD complicated by malperfusion [67]. In-hospital mortality in the traditional cohort was 33.9%, versus 10.2% in the individualized cohort [67]. These data from select expert aortic care centers are promising but expectations should be tempered until multi-center prospective evidence emerges. Guideline-supported practice continues to be traditional central repair first. A suggested algorithm is presented for operative decision making in Fig. 2.

**Fig. 2.**
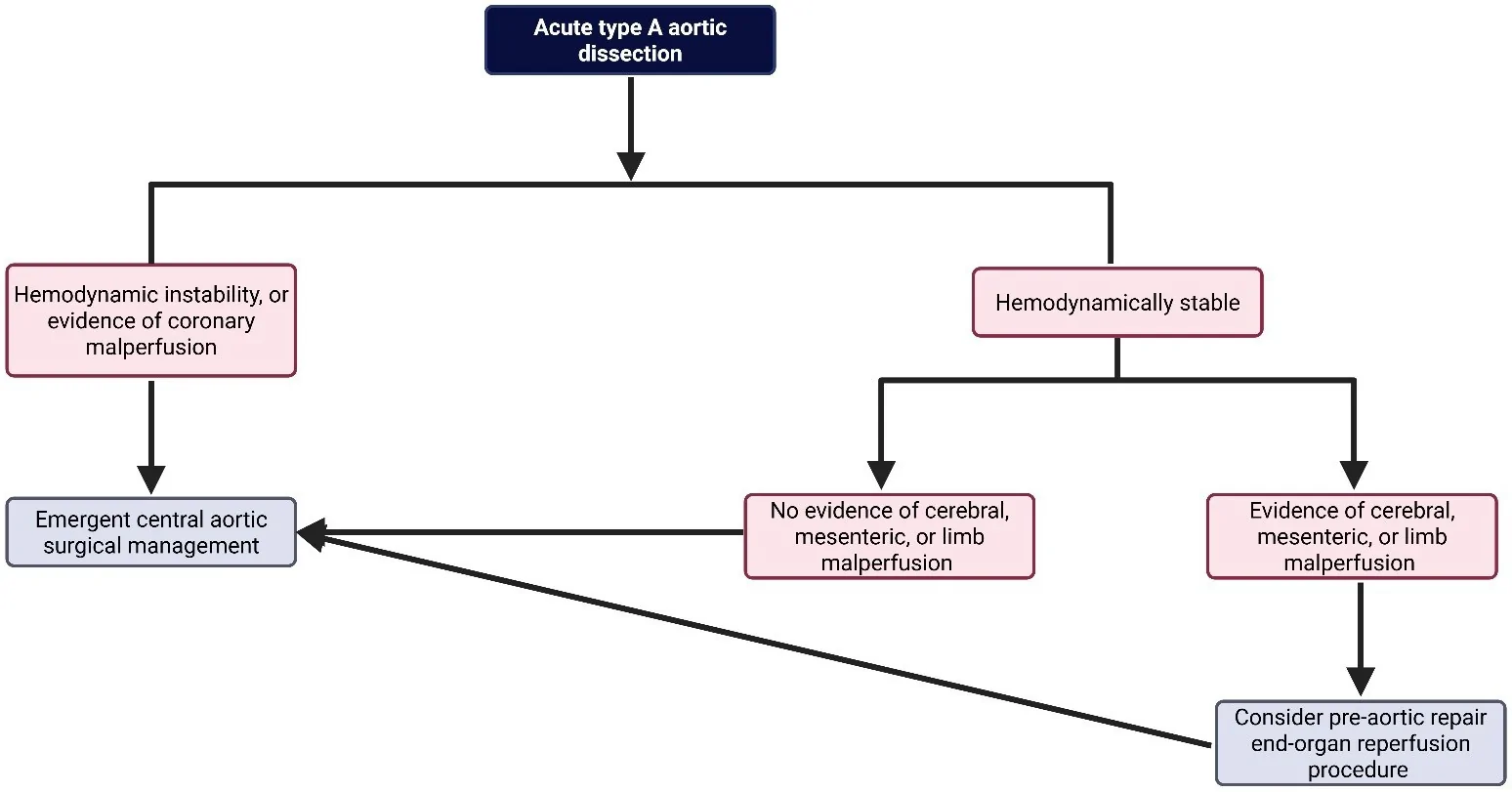
** Surgical decision-making algorithm**. Created in BioRender.

## 12. Cannulation Strategies

Prompt, safe, and reproducible arterial and venous cannulation strategies to establish CPB flow are key to re-establishing true lumen systemic perfusion. Given the variation in anatomic features and presentations of ATAAD, there is not a one-size-fits-all approach to arterial cannulation. Historically, the femoral artery was the preferred site of arterial cannulation given ease of access prior to sternotomy; however, more recently, right axillary, innominate, and central aortic cannulation have gained popularity with evidence of excellent reproducibility and safety [70]. Regardless of arterial cannulation strategy, it is vital to ensure that CPB flow is established in the true lumen.

While its use has declined over time, percutaneous or open femoral artery cannulation can be useful in the setting of hemodynamic instability or when rapid CPB is required, due to the ease of rapid access and exposure, before or during sternotomy. However, the femoral approach carries inherent risks associated with its retrograde perfusion, including the risk of retrograde embolization and false lumen pressurization. A 2021 meta-analysis found that axillary arterial cannulation was associated with significantly reduced odds of stroke when compared with femoral artery cannulation [71]. Despite the risks associated with femoral arterial cannulation for CPB, it remains a relatively fast option for cannulation of patients with hemodynamic instability or challenging dissection anatomy [72,73].

A recent review of International Registry of Acute Aortic Dissection (IRAD) data has identified the axillary artery as the most common arterial cannulation site, with global use of 51% but significant regional variation in practice patterns [73]. The right axillary artery is often favored as a cannulation site given the ability to establish prompt antegrade true lumen flow prior to sternotomy, as well as the ability to provide ACP during circulatory arrest via clamping of the innominate artery [72,74]. The axillary artery is typically approached by a second incision near the deltopectoral groove, with cannulation either via side-graft technique or direct cannulation [75]. Retrospective data comparing direct cannulation of the axillary to side-graft approach demonstrated significantly reduced incidence of cannulation-related complications (stroke and iatrogenic axillary artery dissection) in the side-graft group [76]. Axillary cannulation is a safe and effective option if the dissection extends into the origin of the innominate, but not if it extends into the axillary artery itself. Delivery of ACP via axillary cannulation should be avoided in the case of anomalous right subclavian artery originating from the descending thoracic aorta [75].

Similar to axillary artery cannulation, cannulation of the innominate artery eases the delivery of ACP during hypothermic circulatory arrest, without the need for an additional incision [73]. By clamping the proximal innominate artery, the same cannula can be used to deliver ACP during hypothermic circulatory arrest.

Central aortic cannulation can be performed using the Seldinger technique and utilizing epiaortic ultrasound or TEE, surgeons can carefully deliver a wire and cannula into the true lumen. A single-institution retrospective cohort demonstrated an in-hospital mortality rate of 10%, and an overall stroke rate of 13% [70]. In the case of inadvertent false lumen cannulation, elevated pump pressures will be noted after initiation of CPB [72]. In extreme cases, such as with aortic rupture or circumferential dissection of the ascending aorta, the aorta may be opened and the wire or cannula is introduced under direct visualization (“Samurai” cannulation) [77]. While previously debated, several recent series have demonstrated comparable surgical outcomes among femoral, axillary, direct aortic, and branch vessel cannulation in terms of stroke, mortality and bleeding, while others show increased risk of postoperative stroke with femoral artery cannulation during ATAAD repair with total arch replacement (TAR) with frozen elephant trunk (FET) [78,79,80,81].

## 13. Management of the Proximal Aorta–Aortic Root and Valve Interventions

The aim of surgical repair of ATAAD is resection of the entire ascending aorta and the primary intimal tear, with restoration of true lumen flow [9]. Additionally, any aneurysmal portion of the aorta should be replaced, and aortic valve competence should be ensured.

The dissection must be evaluated pre-operatively to determine the complexity of the arch dissection, considering the time that definitive repair will take, and parsing out which steps must be taken under circulatory arrest, and which can be performed during CPB (often while cooling or rewarming) [9,82,83]. This can guide the order of proximal and distal repair – generally the proximal intervention is completed during CPB (especially during cooling and, if necessary, warming), with distal management undertaken during hypothermic circulatory arrest. An algorithmic approach to the surgical management of ATAAD is presented in Fig. 3.

**Fig. 3.**
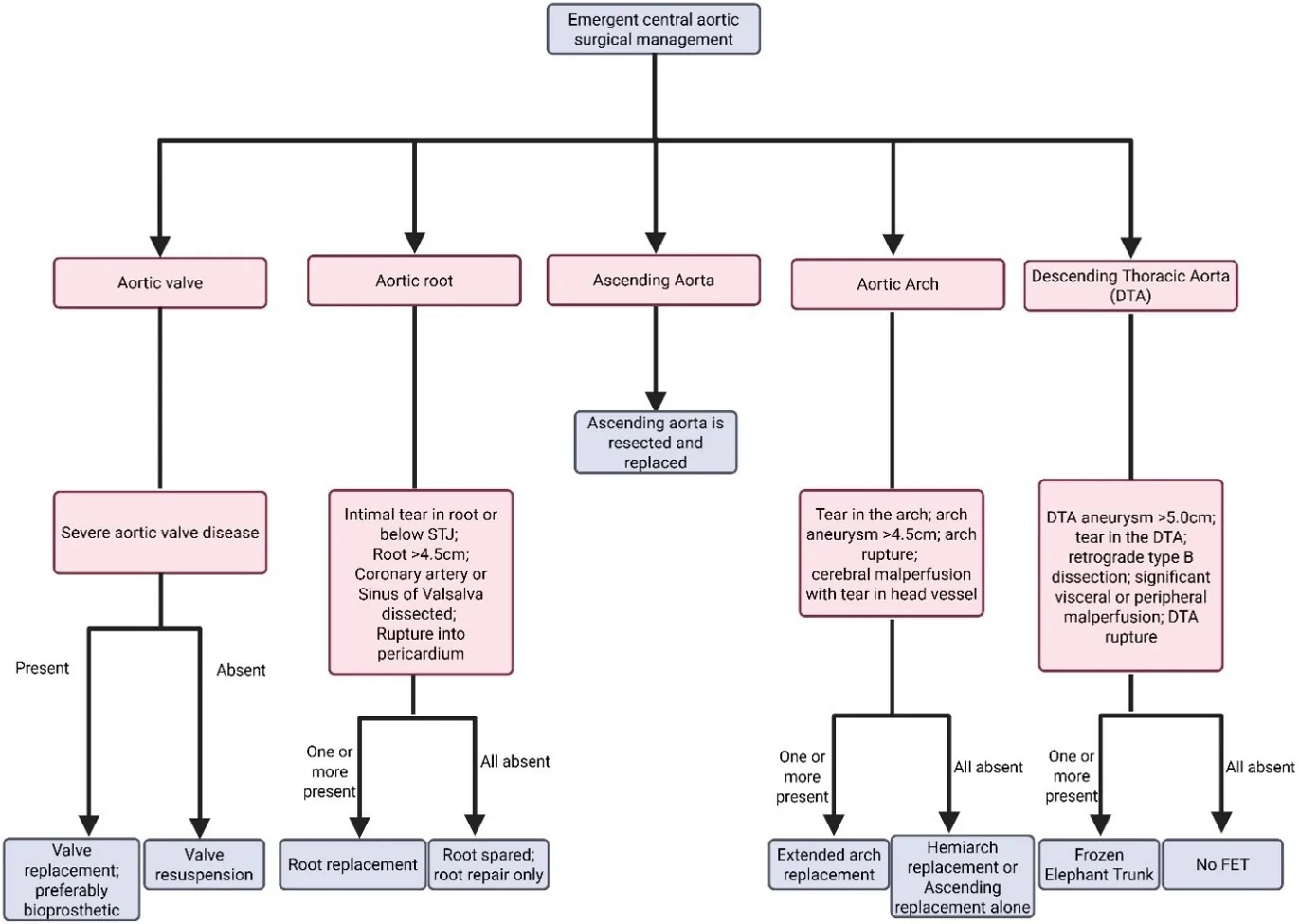
**Central figure: surgical management of the aorta in ATAAD**. ATAAD, Acute type A aortic dissection; STJ, sinotubular junction; FET, frozen elephant trunk. Created in BioRender.

The timing and decision for cross clamp placement is key in managing the proximal aorta. If aortic cross clamping will cause malperfusion, it is unsafe and cannot be performed. In cases of relatively simple arch dissection and clamping will not cause malperfusion, then aortic cross clamping can be performed and repair should begin proximally. In the presence of severe AR, which may manifest with left ventricular distension, aortic cross clamping can assist in preventing retrograde flow from distending the ventricle. However, if cross clamping is deemed unsafe due to high complexity, risk of malperfusion, or concomitant intramural hematoma, then the patient should be cooled and circulation should be arrested without cross clamp placement, and repair should begin in the arch, not proximally [9,82,83].

Proximally, the surgeon must closely evaluate the root for pathology that warrants replacement. If a primary intimal tear is located in the aortic root, below the sinotubular junction (STJ), aortic root replacement is indicated. Additionally, if the aortic root meets size criteria for replacement (typically >4.5 cm), or the coronary arteries are severely dissected, an aortic root replacement should be performed [66].

Next, the aortic valve must be closely inspected. Retrograde propagation of the intimal flap often extends to the level of the root. When this occurs, it is possible to observe catastrophic failure of the aortic valve or valve-supporting structures, either via distortion of root geometry or dynamic prolapse of the intimal flap across the aortic valve. Retrograde propagation, in addition to native valve pathology that is contributing to AR or aortic stenosis, should guide surgical repair strategies [84,85,86,87]. Other key factors that may influence management of the aortic root include compression or deformation of coronary ostia by the false lumen, which can cause coronary malperfusion and myocardial infarction (MI) in up to 5% of patients [88]. Ostial reconstruction or a Cabrol procedure may need to be performed to preserve coronary perfusion [88,89]. If there are any concerns about patency of the coronary arteries, or there are regional wall motion abnormalities on the pre-operative (or post-operative) TEE, this should be addressed with coronary artery bypass before the patient leaves the operating room [88,90].

Proximal aortic rupture into the pericardial space and subsequent cardiac tamponade can also be devastating, increasing the risk of in-hospital mortality among those receiving surgery 2.58-fold [91]. In these cases, the aorta is often friable and malperfused, increasing the technical difficulty of repair, guiding surgeons towards root replacement [66,84,92].

Once the surgeon has decided that the root should be replaced, the next question is how. The main options are valve sparing aortic root replacement (VSARR) and composite valve-graft root replacement. Time from diagnosis to completion of operative intervention is an essential risk factor for post-operative morbidity, and composite valve-graft root replacement offers improved operative speed [28,93]. Recent retrospective cohort studies report that in the setting of ATAAD, the Bentall procedure was associated with shorter CPB times (260 minutes vs 291) and aortic cross clamp times (199 minutes vs 259) when compared to VSARR [94,95,96,97]. If root replacement is indicated in patients who are unstable, Bentall is the preferred method.

Candidates for VSARR should be young, hemodynamically stable, have pliable native aortic valves (bicuspid or tricuspid and without calcification or dissection), and be performed by surgeons who are able to perform VSARR [21,84,85,97]. ATAAD patients with elevated potential for morbidity, such as those with cardiogenic shock, myocardial ischemia, or severe cardiac tamponade, require expeditious definitive repair and therefore composite valve-graft aortic replacement may be more appropriate [9,84,97,98].

VSARR is performed via one of two techniques—the David (reimplantation) procedure or the Yacoub (remodeling) procedure. Given that the Yacoub procedure is often contraindicated in ATAAD, due to its reliance on annular structural integrity, the David procedure is more frequently performed [99]. After VSARR, the native annulus is contained within a Dacron graft, theoretically reducing the risk of future annular dilation [87]. A 2024 analysis of the European registry of ATAAD identified 139 patients who underwent the David procedure with a 10-year mortality rate of 30.1% and a proximal aortic reoperation rate of 3.9% [95]. Other cohorts have reported operative mortality between 0% and 15.9% for VSARR in patients with ATAAD [84,98,99]. The durability, long term quality of the aortic valve, and lack of need for anticoagulation, make VSARR an ideal solution for ATAAD requiring root replacement in young patients, and those with connective tissue disorders [7,9,66,84].

The majority of patients requiring aortic root replacement at the time of ATAAD repair will undergo a composite valve-graft conduit placement, initially described by Bentall and De Bono [100]. Older patients unlikely to benefit from VSARR, those who are unstable at the time of intervention, those with pre-existing valve disease or severe calcification, endocarditis or irreparable root destruction should undergo composite root [97,100,101]. Briefly, the surgeon excises the aortic root including the valve, and implants a composite valve-graft to the left ventricular outflow tract [100]. This process produces a technically simpler approach to aortic root replacement that is less dependent on procedure-specific expertise when compared to valve-sparing root replacement and is more reproducible [84,102]. The Michigan experience comparing the Bentall procedure to VSARR demonstrated that operative mortality outcomes were not significantly different but the composite outcome of MI, stroke, renal failure, and operative mortality significantly favored the David procedure [97]. A single-center study of 314 patients found a short-term mortality rate of the Bentall procedure of 26%, which exceeds the rate of other studies that range from 9.7%–12.9% [95,101]. Furthermore, a 2025 meta-analysis of 4212 aortic root replacement patients found that freedom from reoperation rates were 98.9%, 95.9%, and 90.2% at 1, 5, and 10 years, respectively [103]. Both the Bentall procedure and VSARR are viable options for root replacement, and electing to pursue one or the other must be tailored to fit patient- and surgeon-specific situations, with the emergency setting encouraging many to pursue Bentall given its significantly shorter CPB and aortic cross clamp times.

After evaluation of the aortic root, if the decision is made not to undergo aortic root replacement, aortic root repair should occur. Briefly, the native aortic sinus is preserved, the aortic root is trimmed, false lumen thrombus is removed, the valve is resuspended, and the root is reconstructed and reinforced with inverted intima or felt [66,92,103]. In reapproximating dissected aortic layers, most surgeons have success with neomedia or Teflon felt support, while biologic glue has fallen out of favor due to increased complications of re-intervention [66,92,94,103,104].

A 2014 University of Pennsylvania retrospective study of 489 ATAAD patients analyzed the efficacy of resuspension with felt neomedia repair and found survival rates of 69%, 50%, and 36% at 5, 10, and 15 years, respectively [104]. Impressively, freedom from reoperation at the proximal anastomotic site was 96%, 92%, and 89% at 5, 10, and 15 years, respectively [104]. These data suggest that valve resuspension with adequate aortic wall support remains durable in the long-term. However, leaving the native aortic root tissue that may be weakened by dissection and inflammation can lead to long-term aneurysmal dilation of the root, eventually requiring a complex redo root replacement [92,102,103]. When technically feasible, prioritizing aortic valve repair over replacement is preferred [4,84].

Once the management strategy of the root is selected, a critical decision regarding the management of the valve remains—can the valve be spared or does it need to be replaced? As alluded to above, this often relies on the health and structure of the valve itself [84,85,96,99]. If the valve needs to be replaced, the surgeon needs to decide whether a mechanical or bioprosthetic valve is appropriate. Modern bileaflet mechanical valves have excellent hemodynamics and long-term survival. Yet, mandatory lifelong warfarin use increases the risk of major bleeding, even with lower INR targets (1.5–2.0) [8,86,105]. Bioprosthetic valves avoid lifelong warfarin requirement, but there remains a concern regarding their durability in young patients, as they are more prone to requiring reoperation within 10 to 15 years [106]. Single-institution data comparing mechanical Bentall to bioprosthetic Bentall for ATAAD in patients younger than 65 demonstrated no survival advantage of mech-Bentall compared with bio-Bentall at 10 years, and mech-Bentall was associated with a significantly higher rate of stroke and major bleeding [107]. When the aortic valve cannot be spared, bio-Bentall should be strongly considered in ATAAD, even in young patients.

Aortic root management in ATAAD necessitates rapid evaluation of anatomic, individual, and institutional factors. A reasonable approach involves deciding whether the root needs to be replaced. If the root needs to be replaced—determine which type of replacement is most appropriate (VSARR or Bentall). If the root does not need to be replaced, repair the root and resuspend the valve. If the native valve is damaged beyond repair, then valve replacement and ascending intervention are appropriate.

## 14. Distal Aortic Repair Strategies and Considerations

After assessing the proximal aorta, surgeons must examine the distal aorta on CTA and intra-operatively, evaluating arch diameter, arch vessel anatomy, and location of intimal tear as part of a tear-oriented approach [7,9]. Most patients with ATAAD can be effectively treated with complete resection of the ascending aorta and hemiarch replacement to manage the arch. However, in patients with ATAAD, extended arch intervention is warranted if the primary entry tear is in the arch [9,108]. It may also be warranted if the tear is in the proximal descending aorta, there is brain or peripheral malperfusion, or if there is arch or descending thoracic aneurysm or rupture [108,109,110,111]. Further, extended aortic arch replacement with FET should be considered to provide favorable aortic remodeling and extended aortic arch replacement may be considered in cases of ATAAD in HTAD patients [109,112,113,114,115,116,117].

The fundamental difference between hemiarch replacement and extended arch intervention lies in the extent of aortic tissue replacement, and the consequent complexity of the repair, which is reflected in the operative metrics. Hemiarch replacement involves excising the ascending aorta and extending the graft obliquely into the proximal aortic arch, the lesser curvature, with the distal anastomosis being performed slightly inferior to the origin of the innominate artery [118,119,120]. Conversely, extended arch intervention typically refers to TAR with or without FET. TAR involves the excision of the aortic arch and replacement. This requires the reimplantation of some or all 3 arch vessels onto either a new graft individually (branched graft technique) or all together on shared tissue (island technique) [110,121]. This can be performed with distal anastomosis in zones 1–3, with zone 3 being the most common [83,122]. Typically, a zone 2 anastomosis involves more robust aortic tissue with a better aorta-to-graft size match, while zones 0–1 have improved exposure and are more feasible [83,123,124,125]. With zone 2 arch anastomosis, the left subclavian artery (LSCA) may be covered by concomitant FET or classic elephant trunk (cET). Therefore, the LSCA should either be debranched (locally or extra-anatomically), managed with FET endoprosthesis that includes a subclavian branch, or managed with subsequent TEVAR [83,112,126]. Zone 2 anastomosis is less extensive, results in reduced lower body perfusion arrest time, improved hemostasis, and reduced rate of recurrent laryngeal nerve palsy [122,125]. In the event of hemodynamic instability, or significant comorbidity precluding more complex repair, one can consider less-extensive repair options individualized to the patient. For a proximal tear in the lesser curvature of the arch, with distal extension, one can consider proximal hemiarch replacement with distal TEVAR with partial left subclavian coverage [108,111]. Branched stented anastomosis FET repair, with various configurations selected based on anatomy of dissection, has demonstrated safe and effective early outcomes for ATAAD with multi-segment aortic dissection including a tear in the arch [126]. Finally, while a tear in the arch should be managed with an arch replacement, in extreme cases of hemodynamic instability in relatively poor operative candidates, some may consider hemiarch replacement with simple primary tear repair with felt to perform the fastest possible repair—accepting the increased risk of subsequent aneurysmal degeneration, malperfusion, or rupture with the knowledge that subsequent arch management will be needed in more stable conditions [9]. However, whenever possible—it is essential to attempt to resect or exclude the primary entry tear and perform arch replacement when appropriate [7].

One of the primary concerns with TAR is its potential to increase perioperative morbidity due to increased duration of circulatory arrest time and, traditionally, increased duration with inadequate cerebral perfusion [108]. Meta-analysis of studies comparing TAR versus hemiarch replacement in ATAAD found TAR to be associated with a statistically significant increase in 30-day mortality (OR 1.79), renal failure requiring dialysis (OR 1.34), and stroke (OR 1.49). TAR was also associated with a non-statistically significant increase in long-term mortality (HR 1.25) non-statistically significant decrease in unplanned aortic reoperation (HR 0.53), and a statistically significant decrease in late aortic events (HR 0.53), though subgroup analyses showed higher rates of preoperative malperfusion in the TAR group [111]. A 2021 retrospective cohort study with 365 patients compared the time stratified risks of mortality and neurological injury between the two groups and found that in the post 2009 era, risks were comparable [127]. One study found actuarial survival at 10 years was 49% versus 66% for hemiarch and TAR, respectively, and freedom from reoperation at 10 years was 89% versus 98%, respectively [128]. Similarly, a 2022 meta-analysis of 5243 patients found a significant decrease in the risk of reoperation after 7 years in those who underwent TAR when compared to hemiarch, and TAR yielded a significantly lower risk of mortality at 3 months post-operation [129]. A recent meta-analysis of long-term outcomes suggests significantly improved overall survival with TAR compared with proximal aortic replacement in ATAAD, with significantly lower reoperation rates in patients who underwent TAR [129]. Hemiarch and TAR both play an essential role in the management of ATAAD. The decision to perform a TAR should be individualized to the patient’s situation based on the presence of indication (tear in the arch, arch aneurysm, circumferential arch dissection, or connective tissue disease), patients’ comorbidities and likelihood of living long enough to see a long-term benefit, and the surgeon’s ability to perform a TAR safely [111].

When performing TAR, the order of operations can impact the time of distal organ, and cardiac ischemia and the duration of hypothermia. For example, in branch-first TAR, a trifurcated graft is utilized, and the other head vessels are perfused and provide cardiac perfusion during the third vessel reconstruction. However, others prefer to address the head vessels from distal to proximal during circulatory arrest. Ultimately, the decision of branch vessel order should be individualized to the patient’s anatomy and the dissection pathology [130].

While performing ATAAD repair that does not require extended arch intervention, there exists some debate regarding the role of a true hemiarch replacement under circulatory arrest, versus a “clamp distal”, or an ascending aortic replacement without circulatory arrest [82,118,119,120,131,132]. The conventional approach is to perform the distal anastomosis via an open-distal technique, which permits extension of the resection to the lesser curvature (and extirpation of the cross-clamp site when applicable) and may reduce the risk of false aneurysm formation, false-lumen pressurization, and creation of new entry tears [9,118,119,120]. For most patients, hemiarch replacement remains the preferred strategy. On rare occasions when a patient is unlikely to tolerate circulatory arrest, the operative plan must be highly individualized—for example, limited ascending replacement without circulatory arrest may be chosen as a temporizing measure, with acknowledgment of the increased long-term risk of aneurysmal degeneration or rupture and the likely need for future intervention [7,9,51].

For patients with aneurysmal or dissected descending thoracic aortas, performing a FET will provide an adequate landing zone for staged TEVAR to cover additional tears distal to zone 3, stent open the true lumen in the setting of compression and malperfusion, and encourage healthy aortic remodeling [112,114,115]. The FET technique was initially described using antegrade deployment of a typical TEVAR device into the descending thoracic aorta, and now often utilizes a specialized hybrid FET graft, a composite of a proximal, non-stented fabric tube and a distal, self-expanding stent graft (e.g., Thoraflex Hybrid FET device) [133,134]. The FET evolved from a two-stage cET procedure involving replacement of the ascending aorta and aortic arch with a non-stented vascular graft followed by a second stage in which a new graft was sewn into the free “trunk”, thus extending the repair distally [113,135]. The obliteration of the false lumen promotes its eventual thrombosis, which helps prevent late aneurysmal degeneration [127]. False lumen thrombosis has been reported in up to 92% of patients with ATAAD who underwent FET, with positive remodeling occurring in up to 80% of patients [116].

There are risks associated with performing FET. A 2013 meta-analysis of 8 studies found the overall mortality for patients with ATAAD who underwent TAR with FET was 7.7% (range, 0–18.2%) [109]. A 2025 Taiwanese study of 5769 patients found that when compared to non-FET groups, the in-hospital mortality for those who underwent TAR with FET was significantly higher (22.4% vs 18.0%) [115]. The risk for new onset stroke significantly increases in FET populations [115]. There is also a risk of spinal cord injury/ischemia as a result of using longer, multi-level covered stent grafts [9]. The risk of spinal cord injury is reported to be 2.4% (95% CI, 1.3–4.2%) in patients with ATAAD managed by FET, and this can be significantly decreased by limiting the degree of aortic coverage by the FET to at, or above, T8, or by limiting use of commercial stent grafts to 10-cm length, and avoiding 15-cm length [136,137,138,139]. For postoperative spinal cord ischemia, lumbar cerebrospinal fluid drainage can be used to lower intrathecal pressure and thereby improve spinal cord perfusion pressure; prophylactic use of drains is currently under investigation [140].

In contrast to traditional FET techniques utilizing covered aortic stent grafts, the Ascyrus Medical Dissection Stent (AMDS) is a recently developed hybrid prosthesis meant to augment hemiarch replacement procedures with a proximal cuff of polytetrafluoroethylene (PTFE) and a distal uncovered nitinol stent. This device is primarily indicated for ATAAD with a primary entry tear in the ascending aorta and either clinical or radiographic distal malperfusion. The goal of the AMDS prosthesis is to augment true lumen flow and expansion using an uncovered stent, and seal the distal anastomosis and prevent distal anastomotic new entry tears (DANE) [141,142]. Initial outcomes demonstrate similar operative mortality and in-hospital morbidity, with increased false-lumen thrombosis and decreased rate of DANE, compared to hemiarch alone [143]. The early PERSEVERE results are encouraging: in this particularly sick cohort (ATAAD with a zone 0 tear and malperfusion), the device achieved high technical success and was associated with substantially lower 30-day mortality (9.7% vs 34.6%) and fewer patients experiencing one or more major adverse events (26.9% vs 58.0%) compared with the reference cohort [144].

Additional emerging investigations into the management of the arch include the ARTIZEN trial evaluating the safety and effectiveness of the Arcevo^TM^ (Artivion, Inc) self-expanding aortic stent graft with an integrated stented left subclavian branch preloaded onto a delivery system for use during the FET procedure, preventing coverage of the left subclavian [126,145]. Finally, pre-clinical studies demonstrate the technical feasibility, decreased time for technical anastomosis, and improved anastomotic quality in aortic surgery with aortic end-to-end anastomotic stapler device use [146]. While these developments from select expert aortic care centers for the ARTIZEN trial and use of AMDS are promising, further study is essential before they drive evidence-based guidelines.

## 15. Endovascular Management of ATAAD

While endovascular management of type B aortic dissection is well described, endovascular management of ATAAD is not guideline-directed or the standard of care. Nevertheless, for high-risk patients deemed unsuitable for open repair, there is growing interest in developing and refining endovascular management strategies [147,148,149]. The anatomic feasibility of commercially available stent grafts to treat ATAAD is low. The ascending aorta is frequently dilated and the proximal landing zone short, with the entry tear often very close to the STJ—conditions that make secure sealing difficult and increase the risk of endoleak, coronary encroachment, aortic valve distortion, or compromise of innominate flow [148]. Fenestrated or branched valve-carrying conduits may overcome these problems, creating a more proximal landing zone because they can be anchored in the annulus [150]. Endovascular aortic root repair in the setting of ATAAD has been executed successfully using physician-modified composite grafts [151]. While reviews describe achievable anatomy for endovascular approach and there are case series with reasonable short-term outcomes, the technique remains experimental. Until long-term outcomes are available, and better-fit devices are commercially available, open surgical management will likely be the gold standard supported by guidelines. The ARISE multicenter investigation is evaluating the efficacy of an ascending stent graft for use in patients with ATAAD who are deemed high-risk surgical candidates with amenable anatomy, with promising early results (20% 30-day mortality, 6.7% stroke with a high degree of technical success) [152].

## 16. Organ Protection During Aortic Cross Clamping and Circulatory Arrest

ATAAD has the potential to compromise blood flow to all major organs, including the brain, spinal cord, heart, abdominal viscera, kidneys, and extremities. In addition to restoring true lumen flow, the objective of the operation should be protection and restoration of function of these end-organs.

Primary repair of the dissection—with adjunctive branch-vessel interventions when needed—is required to restore and maintain perfusion to the heart, abdominal viscera, kidneys, and extremities. Strategies for cerebral protection, however, vary substantially because hemiarch replacement and more extensive arch work require a period of circulatory arrest, necessitating additional measures to preserve brain perfusion and metabolism. While visceral organ protection is solely centered on minimizing metabolic demand, cerebral protection during ATAAD repair also relies on alternative perfusion while antegrade aortic blood flow is halted. One of the key decisions in end-organ protection is the degree of hypothermia. Systemic hypothermia reduces end-organ metabolic rate, lowering the oxygen demand of the brain and other ischemic organs and thereby decreasing the risk of organ injury during the period of circulatory arrest required for ATAAD repair [153]. Cardioplegia delivered into the coronary ostia and via a retrograde coronary sinus catheter should be given at regular intervals determined by the cardioplegia solution and institutional protocol with continuous confirmation of adequate cardiac arrest [154]. Deep hypothermic circulatory arrest (DHCA), defined as core body temperature below 20 °C, began as the standard of care for metabolic protection. However, prolonged deeper hypothermia is associated with increased risk of coagulopathy, neurologic dysfunction, and other risks. Therefore, moderate hypothermic circulatory arrest (MHCA), defined as core body temperature between 20.1–28 °C, is becoming increasingly utilized. Based on IRAD data, before 2010, significantly more patients underwent dissection repair using DHCA than MHCA (57.7% vs 42.3%), whereas between 2010 and 2018, patients underwent dissection repair using MHCA significantly more frequently than DHCA (62.5% vs 37.5%) [155]. Recent studies demonstrated that MHCA provides similar visceral end-organ protection to DHCA, and should be preferred for periods of visceral ischemia less than 60 min, as MHCA may be associated with a reduced inflammatory response and reperfusion injury [156].

ACP and/or retrograde cerebral perfusion (RCP) during circulatory arrest is a popular adjunct used to provide cerebral blood flow while the pump is off. The two main methods for perfusion delivery are ACP and RCP. ACP is defined as perfusion that supplies the brain in a normal physiological direction via direct cannulation of the right axillary artery, innominate artery, or carotid arteries, once circulatory arrest has begun. By maintaining forward arterial inflow, ACP preserves oxygen tension, glucose delivery, and acid-base balance within the cerebral microcirculation, thus helping sustain aerobic metabolism and prevent ischemic cytotoxic shift [153,157]. The physiologic orientation of flow allows ACP to match metabolic demand more effectively than cooling alone, which has enabled the use of MHCA for complex aortic surgeries, given that cerebral metabolic suppression from MHCA alone would be insufficient [158].

In RCP, cannulation of the superior vena cava allows oxygenated blood to flow superiorly through the cerebral capillaries and through the arterial system, eventually returning centrally via retrograde flow [159]. Although RCP has demonstrated effectiveness in enhancing cerebral protection and in reducing the rate of embolic events by particulate washout, it demonstrates limited capacity to provide meaningful nutrient flow to the brain. This limitation arises from the venous counterpressure and collapse of venous channels under hypothermic circulatory arrest [158]. A single-center ATAAD series found similar 30-day mortality, new neurological dysfunction, and intensive care unit (ICU) or in-hospital stay between ACP and RCP with a standardized protocol for TAR [160].

In the last 15 years, MHCA, used in conjunction with ACP, has proven to be associated with outcomes comparable to DHCA in several studies. A 2021 IRAD analysis of patients with ATAAD who underwent surgical repair in DHCA vs MHCA showed no significant difference in propensity matched stroke, or short-term mortality [155]. A 2023 randomized clinical trial found that lower ranges of MHCA with ACP provided similar protection to DHCA with ACP as evidenced by statistically equivalent rates and severity of global cognitive ability of patients 4 weeks after aortic repair requiring circulatory arrest [161]. A 2016 study found that MHCA and ACP were associated with a 76.5% significantly decreased risk of postoperative neurologic complications and all-cause mortality when compared to DHCA and RCP [162]. A further topic of investigation is whether unilateral or bilateral ACP is superior. Recent literature has suggested that there is no significant difference between the two ACP techniques as the unilateral method is non-inferior to that of bilateral. Therefore, the unilateral ACP method is recommended as it involves less manipulation of arch branch vessels and is simpler [58,163].

Many studies suggest that DHCA alone is safe for up to 40 minutes, MHCA is safe for up to 20 minutes, and ACP prolongs the duration of safety for both temperature approaches [161,164,165,166,167,168]. Due to the heterogeneity of the results in these studies, neuromonitoring methods have been developed to confirm minimal cerebral activity before circulatory arrest. Identifying and correcting cerebral malperfusion by modifying operative technique was associated with improved neurologic outcomes [169,170]. In practice, temperature management and cerebral perfusion in ATAAD repair are complementary tools that must be used mindfully to match the planned surgery to the protection strategy [9,155,161].

## 17. Postoperative Care

Though the rate of in-hospital mortality for ATAAD has been down-trending from 31% to 22%, likely as a result of down-trending surgical mortality from 25% to 18%, ATAAD continues to be a devastating diagnosis [6]. As such, effective postoperative management of patients recovering from ATAAD is critical for optimizing outcomes. In the immediate post-operative period, patients recover in the intensive care setting. The intensive care setting offers increased nursing and provider attention, such that the likelihood that common yet devastating complications (stroke, mesenteric ischemia, renal failure, bleeding, paralysis, and acute limb ischemia) are found promptly, with the resources necessary to manage them. Similar to the acute medical management of ATAAD, post-operative management of ATAAD involves anti-impulse therapy and ensuring adequate perfusion [4,32,36,171,172]. Postoperative malperfusion is associated with high in-hospital mortality rates [173]. Early neurologic exam can screen for cerebral and spinal cord malperfusion [34,136,137,138].

Bleeding represents a critical post-operative complication that requires early identification and treatment. ATAAD is associated with coagulopathy for a multitude of reasons. Pre-operatively, blood enters the false aortic lumen, which is not covered by endothelium, resulting in massive secretion of tissue factor that results in extensive coagulation activation and hyperfibrinolysis [174]. In the early post-operative period, hypothermia, hemodilution, and administration of heparin all contribute to the coagulopathy experienced by patients. Temperature management, close activated clotting time monitoring and goal-directed therapy, and judicious blood product utilization help optimize coagulopathy in patients with ATAAD [174]. Prospective randomized data demonstrated that thromboelastography-guided transfusion was associated with a decreased use of allogeneic blood units and reduced incidence of massive transfusion compared with other transfusion strategies [174,175]. Rescue therapy for refractory bleeding following ATAAD repair with factor VIII inhibitor bypassing activity (FEIBA) resulted in reduced transfusion requirement in the first 48 hours after surgery and should be considered as an adjunct to thromboelastography-guided transfusion [176].

## 18. Long-Term Management and Follow-Up

After surgical ATAAD management, computed tomographic imaging or magnetic resonance imaging of the aorta with transthoracic echocardiography is recommended before discharge, 1, 6, and 12 months post-dissection, and if stable, annually thereafter [1]. Ideally, this imaging would utilize the same modality and be performed at the same institution for optimal comparison [1]. The goal of this imaging surveillance is to detect enlargement and/or aneurysmal degeneration of the distal aorta in a timely fashion, and monitor visceral vessel dissection. Visceral vessel dissection does not necessarily warrant descending aortic intervention at the time of emergent ATAAD repair, but patients should be followed closely to identify distal aortic aneurysmal degeneration early [177]. Patients who underwent isolated ascending aortic repair remain at risk for progressive enlargement of unrepaired segments of residual dissected aorta and potential growth of non-dissected native aortic segments because of underlying medial degeneration [177]. Were this to occur, it would likely necessitate repeat intervention on the root, arch, or descending aorta [4]. Patients with residual distal dissections after ATAAD repair, especially those with patent distal false lumen and initial very proximal tear, are at risk for aortic growth and reintervention [178]. Those with chronic residual thoracoabdominal dissection and an aneurysm with total aortic diameter of at least 5.5 cm should undergo elective thoracic aortic repair [4].

Care for patients with ATAAD includes screening for HTAD. Indications for HTAD workup include: (1) thoracic aortic disease (TAD) in patients younger than 60 years old, (2) TAD in patients with syndromic features of Marfan syndrome, Loeys-Dietz syndrome, or vascular Ehlers-Danlos syndrome, (3) TAD with a family history of TAD or peripheral/intracranial aneurysms in a first- or second-degree relative, and (4) TAD with a history of unexplained sudden death at a relatively young age in a first- or second-degree relative [4]. All these patients should receive genetic testing for *FBN1*, *LOX*, *COL3A1*, *TGFBR1*, *TGFBR2*, *SMAD3*, *TGFB2*, *ACTA2*, *MYH11*, *MYLK*, and *PRKG1* to identify pathogenic or likely pathogenic variants, and if any are established, it is recommended to counsel patients about their variant, and recommend cascade genetic testing for their family members [179]. If family members have the pathogenic or likely pathogenic variants from the genetic screening—TTE or CT is recommended to visualize the aorta [4]. Any patients with identified genetic variants or who experienced ATAAD should be followed by a multidisciplinary aortic care center [1,4,7,9,10,180].

## 19. Outcomes and Prognosis

As previously mentioned, IRAD data report an overall in-hospital mortality of 10–20% for ATAAD, with a 30% in-hospital mortality rate among patients presenting preoperatively with tamponade, shock, heart failure, stroke, coma, MI, acute renal failure, or mesenteric ischemia [5,6]. Cardiac tamponade, often associated with aortic rupture, has been reported in 8–31% of ATAAD cases [181]. Patients with ATAAD with associated MI experience significantly higher mortality rates (33%) compared to those who do not have MI (15.6%) [182]. Among hospital survivors, 10-year survival after ATAAD repair ranges from about 45–70% across studies, reflecting the high burden of comorbidity and progressive aortic disease. Predictors of poorer long-term outcome include renal dysfunction requiring dialysis, cardiogenic shock or malperfusion at presentation, older age, preoperative frailty or impaired mobility, and lower socioeconomic status [183,184,185,186].

After successful repair of ATAAD, distal aortic pathology remains among the most common causes of late mortality [187,188]. This distal aortic pathology (including aneurysmal degeneration of residual arch dissection) can be associated with the need for distal re-intervention. Single-institution prospective data found that re-operative aortic arch reconstruction following a structured algorithm matching the extent of repair with patient pathology resulted in low rates of in-hospital and 30-day mortality (2.8% and 5.6%, respectively) [189]. When these interventions are performed in a controlled setting, operative outcomes are promising—risk of mortality is around 4–6% for both redo root and redo TAR with FET following ATAAD repair [190,191]. This demonstrates the importance of close follow-up with a multidisciplinary team specializing in the care of patients with aortic disease [192].

## 20. Conclusion

Despite decades of advances in the diagnosis and management of ATAAD, it continues to be associated with significant morbidity and mortality. This review underscores several key themes that improve care of this complex pathology. Optimizing time from presentation to surgery at a high-volume center, individualizing treatment based on malperfusion patterns and surgeon expertise, and employing targeted revascularization strategies in select cases are key to improving outcomes. An algorithmic approach to evaluating ATAAD allows surgeons to individualize the management technique (including cannulation strategy, extent of resection and reconstruction, and end-organ protection techniques) to patient pathology and surgeon expertise. A brief operative video demonstrating the execution of our operative management of ATAAD algorithm (Fig. 3) is shown in **Supplementary Video 1**. There are several promising innovations in development including novel hybrid prostheses and endovascular approaches for select high-risk patients, that have the potential to further improve outcomes. It is essential that we continue to develop and improve tools for managing patients with this complex aortic pathology at aortic centers.

## References

[1] Hiratzka LF, Bakris GL, Beckman JA, Bersin RM, Carr VF, WRITING GROUP MEMBERS (2010). 2010 ACCF/AHA/AATS/ACR/ASA/SCA/SCAI/SIR/STS/SVM guidelines for the diagnosis and management of patients with Thoracic Aortic Disease: a report of the American College of Cardiology Foundation/American Heart Association Task Force on Practice Guidelines, American Association for Thoracic Surgery, American College of Radiology, American Stroke Association, Society of Cardiovascular Anesthesiologists, Society for Cardiovascular Angiography and Interventions, Society of Interventional Radiology, Society of Thoracic Surgeons, and Society for Vascular Medicine. Circulation.

[2] Tsai TT, Nienaber CA, Eagle KA (2005). Acute aortic syndromes. Circulation.

[3] Bonaca MP, O’Gara PT (2014). Diagnosis and management of acute aortic syndromes: dissection, intramural hematoma, and penetrating aortic ulcer. Current Cardiology Reports.

[4] Isselbacher EM, Preventza O, Hamilton Black Iii J, Augoustides JG, Beck AW, Bolen MA (2022). 2022 ACC/AHA Guideline for the Diagnosis and Management of Aortic Disease: A Report of the American Heart Association/American College of Cardiology Joint Committee on Clinical Practice Guidelines. Journal of the American College of Cardiology.

[5] Pape LA, Awais M, Woznicki EM, Suzuki T, Trimarchi S, Evangelista A (2015). Presentation, Diagnosis, and Outcomes of Acute Aortic Dissection: 17-Year Trends From the International Registry of Acute Aortic Dissection. Journal of the American College of Cardiology.

[6] Evangelista A, Isselbacher EM, Bossone E, Gleason TG, Eusanio MD, Sechtem U (2018). Insights From the International Registry of Acute Aortic Dissection: A 20-Year Experience of Collaborative Clinical Research. Circulation.

[7] Czerny M, Grabenwöger M, Berger T, Aboyans V, Della Corte A, Chen EP (2024). EACTS/STS Guidelines for diagnosing and treating acute and chronic syndromes of the aortic organ. European Journal of Cardio-Thoracic Surgery.

[8] Otto CM, Nishimura RA, Bonow RO, Carabello BA, Erwin JP, Gentile F (2021). 2020 ACC/AHA Guideline for the Management of Patients With Valvular Heart Disease: A Report of the American College of Cardiology/American Heart Association Joint Committee on Clinical Practice Guidelines. Circulation.

[9] Malaisrie SC, Szeto WY, Halas M, Girardi LN, Coselli JS, Sundt TM (2021). 2021 The American Association for Thoracic Surgery expert consensus document: Surgical treatment of acute type A aortic dissection. The Journal of Thoracic and Cardiovascular Surgery.

[10] Mazzolai L, Teixido-Tura G, Lanzi S, Boc V, Bossone E, Brodmann M (2024). 2024 ESC Guidelines for the management of peripheral arterial and aortic diseases. European Heart Journal.

[11] Gouveia E Melo R, Mourão M, Caldeira D, Alves M, Lopes A, Duarte A (2022). A systematic review and meta-analysis of the incidence of acute aortic dissections in population-based studies. Journal of Vascular Surgery.

[12] Tanaka Y, Sakata K, Sakurai Y, Yoshimuta T, Morishita Y, Nara S (2016). Prevalence of Type A Acute Aortic Dissection in Patients With Out-Of-Hospital Cardiopulmonary Arrest. The American Journal of Cardiology.

[13] Goel NJ, Kelly JJ, Patrick WL, Zhao Y, Bavaria JE, Ouzounian M (2025). Malperfusion in Patients With Acute Type A Aortic Dissection: A Nationwide Analysis. The Annals of Thoracic Surgery.

[14] Gaines TE, Grimsley LB, Dieter RS, Dieter RA, Dieter RA 3rd (2019). Pathophysiology of Ascending Aortic Aneurysm and Dissection. Diseases of the Aorta.

[15] Mancusi C, Basile C, Fucile I, Palombo C, Lembo M, Buso G (2025). Aortic Remodeling in Patients with Arterial Hypertension: Pathophysiological Mechanisms, Therapeutic Interventions and Preventive Strategies-A Position Paper from the Heart and Hypertension Working Group of the Italian Society of Hypertension. High Blood Pressure & Cardiovascular Prevention: the Official Journal of the Italian Society of Hypertension.

[16] Wu D, Shen YH, Russell L, Coselli JS, LeMaire SA (2013). Molecular mechanisms of thoracic aortic dissection. The Journal of Surgical Research.

[17] Landenhed M, Engström G, Gottsäter A, Caulfield MP, Hedblad B, Newton-Cheh C (2015). Risk profiles for aortic dissection and ruptured or surgically treated aneurysms: a prospective cohort study. Journal of the American Heart Association.

[18] Fletcher AJ, Syed MBJ, Aitman TJ, Newby DE, Walker NL (2020). Inherited Thoracic Aortic Disease: New Insights and Translational Targets. Circulation.

[19] Kamel H, Roman MJ, Pitcher A, Devereux RB (2016). Pregnancy and the Risk of Aortic Dissection or Rupture: A Cohort-Crossover Analysis. Circulation.

[20] Whelan AR, Ringel ME, Shalhub S, Russo ML (2023). Obstetric considerations for aortopathy in pregnancy. Annals of Cardiothoracic Surgery.

[21] Isselbacher EM, Preventza O, Hamilton Black J, Augoustides JG, Beck AW, Bolen MA (2022). 2022 ACC/AHA Guideline for the Diagnosis and Management of Aortic Disease: A Report of the American Heart Association/American College of Cardiology Joint Committee on Clinical Practice Guidelines. Circulation.

[22] Lindley KJ, Bairey Merz CN, Asgar AW, Bello NA, Chandra S, Davis MB (2021). Management of Women With Congenital or Inherited Cardiovascular Disease From Pre-Conception Through Pregnancy and Postpartum: JACC Focus Seminar 2/5. Journal of the American College of Cardiology.

[23] De Bakey ME, Henly WS, Cooley DA, Morris GC, Crawford ES, Beall AC (1965). SURGICAL MANAGEMENT OF DISSECTING ANEURYSMS OF THE AORTA. The Journal of Thoracic and Cardiovascular Surgery.

[24] Sievers HH, Rylski B, Czerny M, Baier ALM, Kreibich M, Siepe M (2020). Aortic dissection reconsidered: type, entry site, malperfusion classification adding clarity and enabling outcome prediction. Interactive CardioVascular and Thoracic Surgery.

[25] Lombardi JV, Hughes GC, Appoo JJ, Bavaria JE, Beck AW, Cambria RP (2020). Society for Vascular Surgery (SVS) and Society of Thoracic Surgeons (STS) Reporting Standards for Type B Aortic Dissections. The Annals of Thoracic Surgery.

[26] Booher AM, Isselbacher EM, Nienaber CA, Trimarchi S, Evangelista A, Montgomery DG (2013). The IRAD classification system for characterizing survival after aortic dissection. The American Journal of Medicine.

[27] Hagan PG, Nienaber CA, Isselbacher EM, Bruckman D, Karavite DJ, Russman PL (2000). The International Registry of Acute Aortic Dissection (IRAD): new insights into an old disease. JAMA.

[28] Matthews CR, Madison M, Timsina LR, Namburi N, Faiza Z, Lee LS (2021). Impact of time between diagnosis to treatment in Acute Type A Aortic Dissection. Scientific Reports.

[29] Acharya M, Mariscalco G (2023). Diagnosis and acute management of type A aortic dissection. The British Journal of Cardiology.

[30] Ko JP, Goldstein JM, Latson LA, Azour L, Gozansky EK, Moore W (2021). Chest CT Angiography for Acute Aortic Pathologic Conditions: Pearls and Pitfalls. Radiographics.

[31] Baliyan V, Parakh A, Prabhakar AM, Hedgire S (2018). Acute aortic syndromes and aortic emergencies. Cardiovascular Diagnosis and Therapy.

[32] Chen SW, Chan YH, Lin CP, Wu VCC, Cheng YT, Chen DY (2021). Association of Long-term Use of Antihypertensive Medications With Late Outcomes Among Patients With Aortic Dissection. JAMA Network Open.

[33] Flower L, Arrowsmith JE, Bewley J, Cook S, Cooper G, Flower J (2023). Management of acute aortic dissection in critical care. Journal of the Intensive Care Society.

[34] Harrington DK, Ranasinghe AM, Shah A, Oelofse T, Bonser RS (2011). Recommendations for haemodynamic and neurological monitoring in repair of acute type a aortic dissection. Anesthesiology Research and Practice.

[35] Roscoe A, Mackay J, Arrowsmith J (2020). Miscellaneous Topics. Core Topics in Cardiac Anaesthesia.

[36] Suzuki T, Isselbacher EM, Nienaber CA, Pyeritz RE, Eagle KA, Tsai TT (2012). Type-Selective Benefits of Medications in Treatment of Acute Aortic Dissection (from the International Registry of Acute Aortic Dissection [IRAD]). The American Journal of Cardiology.

[37] Gilon D, Korach A, Carbone A, Evangelista A, Brinster DR, Estrera AL (2025). Cardiac Tamponade Complicating Type A Acute Aortic Dissection: Insights From 25 Years of Registry Research. JACC. Advances.

[38] Hayashi T, Tsukube T, Yamashita T, Haraguchi T, Matsukawa R, Kozawa S (2012). Impact of controlled pericardial drainage on critical cardiac tamponade with acute type A aortic dissection. Circulation.

[39] Obel LM, Lindholt JS, Lasota AN, Jensen HK, Benhassen LL, Mørkved AL (2022). Clinical Characteristics, Incidences, and Mortality Rates for Type A and B Aortic Dissections: A Nationwide Danish Population-Based Cohort Study From 1996 to 2016. Circulation.

[40] Teurneau-Hermansson K, Ede J, Larsson M, Linton G, von Rosen D, Sjögren J (2024). Mortality after non-surgically treated acute type A aortic dissection is higher than previously reported. European Journal of Cardio-Thoracic Surgery.

[41] Mariscalco G, Maselli D, Zanobini M, Ahmed A, Bruno VD, Benedetto U (2018). Aortic centres should represent the standard of care for acute aortic syndrome. European Journal of Preventive Cardiology.

[42] Andersen ND, Ganapathi AM, Hanna JM, Williams JB, Gaca JG, Hughes GC (2014). Outcomes of acute type a dissection repair before and after implementation of a multidisciplinary thoracic aortic surgery program. Journal of the American College of Cardiology.

[43] Takahashi T, Yoshino H, Shimokawa T, Usui M, Kunihara T, Akutsu K (2025). Interfacility Transfer and Early Mortality in Type A Acute Aortic Dissection: A Multicenter Registry Study. JACC. Asia.

[44] Aggarwal B, Raymond CE, Randhawa MS, Roselli E, Jacob J, Eagleton M (2014). Transfer metrics in patients with suspected acute aortic syndrome. Circulation. Cardiovascular Quality and Outcomes.

[45] Berretta P, Patel HJ, Gleason TG, Sundt TM, Myrmel T, Desai N (2016). IRAD experience on surgical type A acute dissection patients: results and predictors of mortality. Annals of Cardiothoracic Surgery.

[46] Goldstone AB, Chiu P, Baiocchi M, Lingala B, Lee J, Rigdon J (2019). Interfacility Transfer of Medicare Beneficiaries With Acute Type A Aortic Dissection and Regionalization of Care in the United States. Circulation.

[47] Usui M, Yoshino H, Akutsu K, Kunihara T, Shimokawa T, Ogino H (2026). Interfacility Transfer and Mortality in Patients With Type A Acute Aortic Dissection - Analysis of the Tokyo Acute Aortic Super-Network Database. Circulation Reports.

[48] McClure RS, Li W, Gozdyra P, Payne D, Rommens KL, Keir M (2025). Impact of Interhospital Transfer on Mortality for Acute Aortic Dissections. The Annals of Thoracic Surgery.

[49] Bossone E, Eagle KA, Nienaber CA, Trimarchi S, Patel HJ, Gleason TG (2024). Acute Aortic Dissection: Observational Lessons Learned From 11 000 Patients. Circulation. Cardiovascular Quality and Outcomes.

[50] Mehta CK, Chiu S, Hoel AW, Vassallo P, Whippo B, Andrei AC (2023). Implementation of a direct-to-operating room aortic emergency transfer program: Expedited management of type A aortic dissection. The American Journal of Emergency Medicine.

[51] Czerny M, Schmidli J, Adler S, van den Berg JC, Bertoglio L, Carrel T (2019). Editor's Choice - Current Options and Recommendations for the Treatment of Thoracic Aortic Pathologies Involving the Aortic Arch: An Expert Consensus Document of the European Association for Cardio-Thoracic Surgery (EACTS) & the European Society for Vascular Surgery (ESVS). European Journal of Vascular and Endovascular Surgery.

[52] Patrick WL, Yarlagadda S, Bavaria JE, Kelly JJ, Kalva S, Grimm JC (2023). The Penn Classification System for Malperfusion in Acute Type A Dissection: A 25-Year Experience. The Annals of Thoracic Surgery.

[53] Geirsson A, Szeto WY, Pochettino A, McGarvey ML, Keane MG, Woo YJ (2007). Significance of malperfusion syndromes prior to contemporary surgical repair for acute type A dissection: outcomes and need for additional revascularizations. European Journal of Cardio-Thoracic Surgery.

[54] Crawford TC, Beaulieu RJ, Ehlert BA, Ratchford EV, Black JH (2016). Malperfusion syndromes in aortic dissections. Vascular Medicine.

[55] Brown JA, Aranda-Michel E, Navid F, Serna-Gallegos D, Thoma F, Sultan I (2024). Outcomes of emergency surgery for acute type A aortic dissection complicated by malperfusion syndrome. The Journal of Thoracic and Cardiovascular Surgery.

[56] Charlton-Ouw KM, Sritharan K, Leake SS, Sandhu HK, Miller CC, Azizzadeh A (2013). Management of limb ischemia in acute proximal aortic dissection. Journal of Vascular Surgery.

[57] Chen YF, Chien TM, Yu CP, Ho KJ, Wen H, Li WY (2013). Acute aortic dissection type A with acute coronary involvement: a novel classification. International Journal of Cardiology.

[58] Pacini D, Murana G, Di Marco L, Berardi M, Mariani C, Coppola G (2018). Cerebral perfusion issues in type A aortic dissection. Journal of Visualized Surgery.

[59] Czerny M, Siepe M, Beyersdorf F, Feisst M, Gabel M, Pilz M (2020). Prediction of mortality rate in acute type A dissection: the German Registry for Acute Type A Aortic Dissection score. European Journal of Cardio-Thoracic Surgery.

[60] Pacini D, Leone A, Belotti LMB, Fortuna D, Gabbieri D, Zussa C (2013). Acute type A aortic dissection: significance of multiorgan malperfusion. European Journal of Cardio-Thoracic Surgery.

[61] Zindovic I, Sjögren J, Bjursten H, Danielsson E, Ingemansson R, Nozohoor S (2015). Impact of Hemodynamic Instability and Organ Malperfusion in Elderly Surgical Patients Treated for Acute Type A Aortic Dissection. Journal of Cardiac Surgery.

[62] Luo JC, Zhong J, Duan WX, Tu GW, Wang CS, Sun YX (2020). Early risk stratification of acute type A aortic dissection: development and validation of a predictive score. Cardiovascular Diagnosis and Therapy.

[63] Leshnower BG, Keeling WB, Duwayri YM, Jordan WD, Chen EP (2019). The “thoracic endovascular aortic repair-first” strategy for acute type A dissection with mesenteric malperfusion: Initial results compared with conventional algorithms. The Journal of Thoracic and Cardiovascular Surgery.

[64] Yang B, Rosati CM, Norton EL, Kim KM, Khaja MS, Dasika N (2018). Endovascular Fenestration/Stenting First Followed by Delayed Open Aortic Repair for Acute Type A Aortic Dissection With Malperfusion Syndrome. Circulation.

[65] Sugiyama K, Watanuki H, Okada M, Futamura Y, Wakayama R, Matsuyama K (2020). Revascularization-first strategy in acute aortic dissection with mesenteric malperfusion. Journal of Cardiac Surgery.

[66] Yang B, Norton EL, Hobbs R, Farhat L, Wu X, Hornsby WE (2019). Short- and long-term outcomes of aortic root repair and replacement in patients undergoing acute type A aortic dissection repair: Twenty-year experience. The Journal of Thoracic and Cardiovascular Surgery.

[67] Chen S, Peng H, Zhuang H, Wang J, Yan P, Zhang W (2025). Optimized strategy to improve the outcomes of acute type A aortic dissection with malperfusion syndrome. The Journal of Thoracic and Cardiovascular Surgery.

[68] He K, Qin X, Li M, Bian L, Yue H, Liang W (2024). Two surgical strategies (early carotid reperfusion vs. Central aortic repair-first) of acute type a aortic dissection complicated with cerebral malperfusion syndrome: a meta-analysis and systematic review. BMC Cardiovascular Disorders.

[69] Ebe R, Nomura Y, Mizobe T, Tanaka H (2026). Carotid Artery Stenting Before Aortic Repair for Acute Type A Aortic Dissection Complicated by Cerebral Malperfusion. The Annals of Thoracic Surgery.

[70] Kreibich M, Chen Z, Rylski B, Bavaria JE, Brown CR, Branchetti E (2019). Outcome after aortic, axillary, or femoral cannulation for acute type A aortic dissection. The Journal of Thoracic and Cardiovascular Surgery.

[71] Hussain A, Uzzaman M, Mohamed S, Khan F, Butt S, Khan H (2021). Femoral versus axillary cannulation in acute type A aortic dissections: A meta-analysis. Journal of Cardiac Surgery.

[72] Xia Q, Cao Y, Xie B, Qiu D, Deng L, Wang M (2021). Cannulation strategies in type A aortic dissection: a novel insight narrative review. Journal of Thoracic Disease.

[73] Elbatarny M, Trimarchi S, Korach A, Di Eusanio M, Pacini D, Bekeredjian R (2024). Axillary vs Femoral Arterial Cannulation in Acute Type A Dissection: International Multicenter Data. The Annals of Thoracic Surgery.

[74] Benedetto U, Sinha S, Dimagli A, Cooper G, Mariscalco G, Uppal R (2021). Decade-long trends in surgery for acute Type A aortic dissection in England: A retrospective cohort study. The Lancet Regional Health. Europe.

[75] Sabik JF, Nemeh H, Lytle BW, Blackstone EH, Gillinov AM, Rajeswaran J (2004). Cannulation of the axillary artery with a side graft reduces morbidity. The Annals of Thoracic Surgery.

[76] Puiu PC, Pingpoh C, Beyersdorf F, Czerny M, Keyl C, Kreibich M (2021). Direct Versus Side Graft Cannulation From the Right Axillary Artery in Thoracic Aortic Surgery. The Annals of Thoracic Surgery.

[77] Kitamura T, Nie M, Horai T, Miyaji K (2017). Direct True Lumen Cannulation (“Samurai” Cannulation) for Acute Stanford Type A Aortic Dissection. The Annals of Thoracic Surgery.

[78] Wang X, Liu N, Wang H, Liu Y, Sun L, Zhu J (2023). Femoral artery cannulation increases the risk of postoperative stroke in patients with acute DeBakey I aortic dissection. The Journal of Thoracic and Cardiovascular Surgery.

[79] Lemaire A, Levine D, Chao J, Sabatino ME, Ikegami H, Takebe M (2023). Does arterial cannulation site for aortic dissection repair impact surgical outcomes?. Journal of Thoracic Disease.

[80] Norton EL, Kim KM, Fukuhara S, Naeem A, Wu X, Patel HJ (2022). Aortic and arch branch vessel cannulation in acute type A aortic dissection repair. JTCVS Techniques.

[81] Naito N, Takagi H (2025). Meta-analysis of central and peripheral cannulation for type A aortic dissection. Perfusion.

[82] Stamou SC, Kouchoukos NT, Hagberg RC, Khabbaz KR, Robicsek F, Nussbaum M (2011). Does the technique of distal anastomosis influence clinical outcomes in acute type A aortic dissection?. Interactive Cardiovascular and Thoracic Surgery.

[83] Kang JJH, Moon MC (2024). Total Arch Replacement Operative Techniques. CJC Open.

[84] Leshnower BG, Farrington WJ, Huckaby LV, Keeling WB, Zellner AB, Chen EP (2025). Long-term Results of Valve-Sparing Aortic Root Replacement in Acute Type A Aortic Dissection. The Annals of Thoracic Surgery.

[85] Levine D, Patel P, Zhao Y, Childress P, Chung M, Leshnower BG (2023). Bicuspid aortic valve durability with valve-sparing aortic root replacement: comparison to tricuspid valve. European Journal of Cardio-Thoracic Surgery.

[86] Leviner DB, Abraham D, Ronai T, Sharoni E (2024). Mechanical Valves: Past, Present, and Future-A Review. Journal of Clinical Medicine.

[87] David TE, Feindel CM (1992). An aortic valve-sparing operation for patients with aortic incompetence and aneurysm of the ascending aorta. The Journal of Thoracic and Cardiovascular Surgery.

[88] Ichihara S, Saito Y, Hashimoto O, Sasaki H, Yumoto K, Oshima S (2023). Timing of Myocardial Infarction Diagnosis in Type A Acute Aortic Dissection and Coronary Artery Involvement. JACC. Asia.

[89] Alaivi YT, Adel SMH, Heidari A, Akiash N, Jorfi F (2025). Cabrol Procedure in Complex Aortic Root Reconstruction: A Case Series of Three Young Patients With Acute Aortic Syndrome. Clinical Case Reports.

[90] Ogami T, Serna-Gallegos D, Yousef S, Brown JA, Thoma FW, Subramaniam K (2024). The Clinical Significance of Unplanned Coronary Artery Bypass Grafting in Aortic Root Replacement. Journal of Cardiothoracic and Vascular Anesthesia.

[91] Harris KM, Nienaber CA, Peterson MD, Woznicki EM, Braverman AC, Trimarchi S (2022). Early Mortality in Type A Acute Aortic Dissection: Insights From the International Registry of Acute Aortic Dissection. JAMA Cardiology.

[92] Arabkhani B, Verhoef J, Tomšič A, van Brakel TJ, Hjortnaes J, Klautz RJM (2023). The Aortic Root in Acute Type A Dissection: Repair or Replace?. The Annals of Thoracic Surgery.

[93] Nienaber CA, Kische S, Rousseau H, Eggebrecht H, Rehders TC, Kundt G (2013). Endovascular repair of type B aortic dissection: long-term results of the randomized investigation of stent grafts in aortic dissection trial. Circulation. Cardiovascular Interventions.

[94] Chiu P, Trojan J, Tsou S, Goldstone AB, Woo YJ, Fischbein MP (2018). Limited root repair in acute type A aortic dissection is safe but results in increased risk of reoperation. The Journal of Thoracic and Cardiovascular Surgery.

[95] Biancari F, Mastroiacovo G, Rinaldi M, Ferrante L, Mäkikallio T, Juvonen T (2024). The David Versus the Bentall Procedure for Acute Type A Aortic Dissection. Journal of Cardiovascular Development and Disease.

[96] Formica F, Gallingani A, D’Alessandro S, Tuttolomondo D, Hernandez-Vaquero D, Singh G (2025). Long-term outcomes comparison of Bentall-De Bono-versus valve-sparing aortic root replacement: An updated systematic review and reconstructed time-to-event meta-analysis. International Journal of Cardiology.

[97] Yang B, Patel HJ, Sorek C, Hornsby WE, Wu X, Ward S (2018). Sixteen-Year Experience of David and Bentall Procedures in Acute Type A Aortic Dissection. The Annals of Thoracic Surgery.

[98] Iannacone EM, Lau C, Jr Soletti G, Olaria RP, Harik L, Dimagli A (2023). Aortic valve-sparing root replacement or Bentall?. Annals of Cardiothoracic Surgery.

[99] Rahnavardi M, Yan TD, Bannon PG, Wilson MK (2011). Aortic valve-sparing operations in aortic root aneurysms: remodeling or reimplantation?. Interactive Cardiovascular and Thoracic Surgery.

[100] Bentall H, De Bono A (1968). A technique for complete replacement of the ascending aorta. Thorax.

[101] Khachatryan Z, Leontyev S, Magomedov K, Haunschild J, Holzhey DM, Misfeld M (2021). Management of aortic root in type A dissection: Bentall approach. Journal of Cardiac Surgery.

[102] Magouliotis DE, Sicouri S, Baudo M, Cabrucci F, Yamashita Y, Ramlawi B (2025). Comparative outcomes of aortic root preservation versus root replacement for acute type A aortic dissection: balancing survival, durability, and reoperation. Hellenic Journal of Cardiology.

[103] Zhang Y, Lin H, Li Y, Dai X, Pan D, Yu Z (2025). Aortic root replacement versus preservation in acute type A aortic dissection repair: meta-analysis of reconstructed time-to-event data. Journal of Cardiothoracic Surgery.

[104] Rylski B, Bavaria JE, Milewski RK, Vallabhajosyula P, Moser W, Kremens E (2014). Long-term results of neomedia sinus valsalva repair in 489 patients with type A aortic dissection. The Annals of Thoracic Surgery.

[105] Eikelboom JW, Connolly SJ, Brueckmann M, Granger CB, Kappetein AP, Mack MJ (2013). Dabigatran versus warfarin in patients with mechanical heart valves. The New England Journal of Medicine.

[106] Goldstone AB, Chiu P, Baiocchi M, Lingala B, Patrick WL, Fischbein MP (2017). Mechanical or Biologic Prostheses for Aortic-Valve and Mitral-Valve Replacement. The New England Journal of Medicine.

[107] Catalano MA, Toubat O, Gillinov L, Lawrence KM, Zhao Y, Kelly JJ (2025). Management of the dissected aortic root in young patients: A propensity score-matched analysis of mechanical versus bioprosthetic aortic root replacement. JTCVS Open.

[108] Poon SS, Theologou T, Harrington D, Kuduvalli M, Oo A, Field M (2016). Hemiarch versus total aortic arch replacement in acute type A dissection: a systematic review and meta-analysis. Annals of Cardiothoracic Surgery.

[109] Tian DH, Wan B, Di Eusanio M, Black D, Yan TD (2013). A systematic review and meta-analysis on the safety and efficacy of the frozen elephant trunk technique in aortic arch surgery. Annals of Cardiothoracic Surgery.

[110] Griepp RB, Stinson EB, Hollingsworth JF, Buehler D (1975). Prosthetic replacement of the aortic arch. The Journal of Thoracic and Cardiovascular Surgery.

[111] Hage F, Atarere J, Anteby R, Hage A, Malik MI, Boodhwani M (2024). Total Arch vs Hemiarch Repair in Acute Type A Aortic Dissection: Systematic Review and Meta-Analysis of Comparative Studies. CJC Open.

[112] Roselli EE, Isabella MA (2013). Frozen Elephant Trunk Procedure. Operative Techniques in Thoracic and Cardiovascular Surgery.

[113] Inoue Y, Matsuda H, Omura A, Seike Y, Uehara K, Sasaki H (2019). Comparative study of the frozen elephant trunk and classical elephant trunk techniques to supplement total arch replacement for acute type A aortic dissection†. European Journal of Cardio-Thoracic Surgery.

[114] Acharya M, Sherzad H, Bashir M, Mariscalco G (2022). The frozen elephant trunk procedure: indications, outcomes and future directions. Cardiovascular Diagnosis and Therapy.

[115] Chiu PH, Cheng YT, Chang FC, Chen CY, Chan YH, Wu VCC (2026). Outcomes of Frozen Elephant Trunk for Type A Acute Aortic Dissection: A Nationwide, Population Based Study. European Journal of Vascular and Endovascular Surgery.

[116] Dohle DS, Tsagakis K, Janosi RA, Benedik J, Kühl H, Penkova L (2016). Aortic remodelling in aortic dissection after frozen elephant trunk†. European Journal of Cardio-Thoracic Surgery.

[117] Chen Y, Ma WG, Zhi AH, Lu L, Zheng J, Zhang W (2019). Fate of distal aorta after frozen elephant trunk and total arch replacement for type A aortic dissection in Marfan syndrome. The Journal of Thoracic and Cardiovascular Surgery.

[118] Malvindi PG, Modi A, Miskolczi S, Kaarne M, Velissaris T, Barlow C (2016). Open and closed distal anastomosis for acute type A aortic dissection repair. Interactive Cardiovascular and Thoracic Surgery.

[119] Geirsson A, Shioda K, Olsson C, Ahlsson A, Gunn J, Hansson EC (2019). Differential outcomes of open and clamp-on distal anastomosis techniques in acute type A aortic dissection. The Journal of Thoracic and Cardiovascular Surgery.

[120] Pupovac SS, Jarral OA, Trimarchi S, Sultan I, Leshnower B, Desai ND (2026). Clamp On vs Off Impact of Distal Anastomotic Technique During Ascending Aortic Replacement in Acute Type A Aortic Dissection: IRAD Insights. Interdisciplinary Cardiovascular and Thoracic Surgery.

[121] Spielvogel D, Goldberg JB, Kai M, Malekan R, Lansman SL (2019). Aortic Arch Replacement With a Trifurcated Graft. Operative Techniques in Thoracic and Cardiovascular Surgery.

[122] Arakawa M, Akiyoshi K, Kitada Y, Miyagawa A, Okamura H (2025). Comparison between Zone 2 and Zone 3 distal anastomoses for aortic arch replacement in terms of invasiveness. General Thoracic and Cardiovascular Surgery.

[123] Desai ND, Hoedt A, Wang G, Szeto WY, Vallabhajosyula P, Reinke M (2018). Simplifying aortic arch surgery: open zone 2 arch with single branched thoracic endovascular aortic repair completion. Annals of Cardiothoracic Surgery.

[124] Gottardi R, Voetsch A, Krombholz-Reindl P, Winkler A, Steindl J, Dinges C (2020). Comparison of the conventional frozen elephant trunk implantation technique with a modified implantation technique in zone 1. European Journal of Cardio-Thoracic Surgery.

[125] Appoo JJ, Gregory AJ, Fichadiya A, Kotha VK, Herget EJ (2017). Zone 2 Arch Replacement and Staged Thoracic Endovascular Aortic Repair for Acute Type A Aortic Dissection. The Annals of Thoracic Surgery.

[126] Roselli EE, Vargo PR, Bakaeen F, Koprivanac M, Burns D, Kuramochi Y (2024). Branched stented anastomosis frozen elephant trunk repair: Early results from a physician-sponsored investigational device exemption study. The Journal of Thoracic and Cardiovascular Surgery.

[127] Ok YJ, Kang SR, Kim HJ, Kim JB, Choo SJ (2021). Comparative outcomes of total arch versus hemiarch repair in acute DeBakey type I aortic dissection: the impact of 21 years of experience. European Journal of Cardio-Thoracic Surgery.

[128] Vendramin I, Piani D, Lechiancole A, Sponga S, Di Nora C, Londero F (2022). Hemiarch Versus Arch Replacement in Acute Type A Aortic Dissection: Is the Occam’s Razor Principle Applicable?. Journal of Clinical Medicine.

[129] Sá MP, Jacquemyn X, Tasoudis PT, Van den Eynde J, Erten O, Sicouri S (2022). Long-term outcomes of total arch replacement versus proximal aortic replacement in acute type A aortic dissection: Meta-analysis of Kaplan-Meier-derived individual patient data. Journal of Cardiac Surgery.

[130] Preventza O, Coselli JS (2020). Commentary: Aortic arch repair: The patient’s anatomy and the surgeon’s knowledge matter. JTCVS Techniques.

[131] Danner BC, Natour E, Horst M, Dikov V, Ghosh PK, Dapunt OE (2007). Comparison of operative techniques in acute type A aortic dissection performing the distal anastomosis. Journal of Cardiac Surgery.

[132] Lai DT, Robbins RC, Mitchell RS, Moore KA, Oyer PE, Shumway NE (2002). Does profound hypothermic circulatory arrest improve survival in patients with acute type a aortic dissection?. Circulation.

[133] Shrestha M, Kaufeld T, Beckmann E, Fleissner F, Umminger J, Abd Alhadi F (2016). Total aortic arch replacement with a novel 4-branched frozen elephant trunk prosthesis: Single-center results of the first 100 patients. The Journal of Thoracic and Cardiovascular Surgery.

[134] Shrestha M, Pichlmaier M, Martens A, Hagl C, Khaladj N, Haverich A (2013). Total aortic arch replacement with a novel four-branched frozen elephant trunk graft: first-in-man results. European Journal of Cardio-Thoracic Surgery.

[135] Velasquez CA, Zafar MA, Saeyeldin A, Bin Mahmood SU, Brownstein AJ, Erben Y (2017). Two-Stage Elephant Trunk approach for open management of distal aortic arch and descending aortic pathology in patients with Marfan syndrome. Annals of Cardiothoracic Surgery.

[136] Rezaei Y, Bashir M, Mousavizadeh M, Daliri M, Aljadayel HA, Mohammed I (2021). Frozen elephant trunk in total arch replacement: A systematic review and meta-analysis of outcomes and aortic proximalization. Journal of Cardiac Surgery.

[137] Okita Y (2020). Frozen elephant trunk with Frozenix prosthesis. Annals of Cardiothoracic Surgery.

[138] Katayama K, Uchida N, Katayama A, Takahashi S, Takasaki T, Kurosaki T (2015). Multiple factors predict the risk of spinal cord injury after the frozen elephant trunk technique for extended thoracic aortic disease. European Journal of Cardio-Thoracic Surgery.

[139] Preventza O, Liao JL, Olive JK, Simpson K, Critsinelis AC, Price MD (2020). Neurologic complications after the frozen elephant trunk procedure: A meta-analysis of more than 3000 patients. The Journal of Thoracic and Cardiovascular Surgery.

[140] Zhou C, Hou B, Zhang K, Gao S, Cao F, Ji Y (2025). Protective Effect on Spinal Cord Injury of Prophylactic Cerebrospinal Fluid Drainage in Extensive Aortic Arch Repair for Type A Aortic Dissection: A Retrospective Cohort Study. Journal of the American Heart Association.

[141] Pitts L, Moon MC, Luehr M, Kofler M, Montagner M, Sündermann S (2024). The Ascyrus Medical Dissection Stent: A One-Fits-All Strategy for the Treatment of Acute Type A Aortic Dissection?. Journal of Clinical Medicine.

[142] Catalano MA, Brown CR, Fukuhara S, Moon MC, Szeto WY (2026). Expert Opinion: Where Does the Novel Hybrid Aortic Prosthesis Fit Into the Management of the Aortic Arch in DeBakey Type I Aortic Dissections?. Seminars in Thoracic and Cardiovascular Surgery.

[143] White A, Elfaki L, O’Brien D, Manikala V, Bozso S, Ouzounian M (2024). The Use of the Ascyrus Medical Dissection Stent in Acute Type A Aortic Dissection Repair Reduces Distal Anastomotic New Entry Tear. The Canadian Journal of Cardiology.

[144] Szeto WY, Fukuhara S, Fleischman F, Sultan I, Brinkman W, Arnaoutakis G (2025). A novel hybrid prosthesis for open repair of acute DeBakey type I dissection with malperfusion: Early results from the PERSEVERE trial. The Journal of Thoracic and Cardiovascular Surgery.

[145] (2025). Clinical Study to Evaluate the Safety and Effectiveness of Arcevo LSA (ARTIZEN). https://clinicaltrials.gov/study/NCT07089576.

[146] Osorio-Jaramillo E, El-Nashar J, Kainuma S, Unger E, Schreiner W, El Din M (2026). A Novel Aortic End-to-End Anastomotic Stapler Device-Results of a Human Cadaver Study. European Journal of Cardio-Thoracic Surgery.

[147] Wisneski AD, Kumar V, Vartanian SM, Oskowitz AZ (2022). Toward endovascular treatment of type A aortic dissection with smaller landing zones and more patient eligibility. Journal of Vascular Surgery.

[148] Kern M, Hauck SR, Dachs TM, Haider L, Stelzmüller ME, Ehrlich M (2023). Endovascular repair in type A aortic dissection: Anatomical candidacy for currently manufactured stent grafts and conceptual valve-carrying devices for an Endo-Bentall procedure. European Journal of Cardio-Thoracic Surgery.

[149] Roselli EE, Hasan SM, Idrees JJ, Aftab M, Eagleton MJ, Menon V (2017). Inoperable patients with acute type A dissection: are they candidates for endovascular repair?. Interactive Cardiovascular and Thoracic Surgery.

[150] Kreibich M, Rylski B, Beyersdorf F, Siepe M, Czerny M (2021). Endo-Bentall for proximal aortic dissection: from conception to application. Asian Cardiovascular & Thoracic Annals.

[151] Ghoreishi M, Chahal D, Shah A, Kang J, Hirsch J, Tran D (2023). First-in-Human Endovascular Aortic Root Repair (Endo-Bentall) for Acute Type A Dissection. Circulation. Cardiovascular Interventions.

[152] Roselli EE, Atkins MD, Brinkman W, Coselli J, Desai N, Estrera A (2023). ARISE: First-In-Human Evaluation of a Novel Stent Graft to Treat Ascending Aortic Dissection. Journal of Endovascular Therapy.

[153] Hoffman WE, Charbel FT, Munoz L, Ausman JI (1998). Comparison of brain tissue metabolic changes during ischemia at 35 degrees and 18 degrees C. Surgical Neurology.

[154] Hussain ST, Svensson LG (2016). Surgical techniques in type A dissection. Annals of Cardiothoracic Surgery.

[155] Pupovac SS, Hemli JM, Bavaria JE, Patel HJ, Trimarchi S, Pacini D (2021). Moderate Versus Deep Hypothermia in Type A Acute Aortic Dissection Repair: Insights from the International Registry of Acute Aortic Dissection. The Annals of Thoracic Surgery.

[156] Pacini D, Pantaleo A, Di Marco L, Leone A, Barberio G, Murana G (2014). Visceral organ protection in aortic arch surgery: safety of moderate hypothermia. European Journal of Cardio-Thoracic Surgery.

[157] Liang MY, Tang ZX, Chen GX, Rong J, Yao JP, Chen Z (2014). Is selective antegrade cerebral perfusion superior to retrograde cerebral perfusion for brain protection during deep hypothermic circulatory arrest? Metabolic evidence from microdialysis. Critical Care Medicine.

[158] Montisci A, Maj G, Cavozza C, Audo A, Benussi S, Rosati F (2023). Cerebral Perfusion and Neuromonitoring during Complex Aortic Arch Surgery: A Narrative Review. Journal of Clinical Medicine.

[159] Fernández Suárez FE, Fernández Del Valle D, González Alvarez A, Pérez-Lozano B (2017). Intraoperative care for aortic surgery using circulatory arrest. Journal of Thoracic Disease.

[160] Svensson LG, Blackstone EH, Apperson-Hansen C, Ruggieri PM, Ainkaran P, Naugle RI (2015). Implications from neurologic assessment of brain protection for total arch replacement from a randomized trial. The Journal of Thoracic and Cardiovascular Surgery.

[161] Hughes GC, Chen EP, Browndyke JN, Szeto WY, DiMaio JM, Brinkman WT (2024). Cognitive Effects of Body Temperature During Hypothermic Circulatory Arrest Trial (GOT ICE): A Randomized Clinical Trial Comparing Outcomes After Aortic Arch Surgery. Circulation.

[162] Perreas K, Samanidis G, Thanopoulos A, Georgiopoulos G, Antoniou T, Khoury M (2016). Antegrade or Retrograde Cerebral Perfusion in Ascending Aorta and Hemiarch Surgery? A Propensity-Matched Analysis. The Annals of Thoracic Surgery.

[163] Song SJ, Kim WK, Kim TH, Song SW (2022). Unilateral versus bilateral antegrade cerebral perfusion during surgical repair for patients with acute type A aortic dissection. JTCVS Open.

[164] Gupta P, Harky A, Jahangeer S, Adams B, Bashir M (2018). Varying Evidence on Deep Hypothermic Circulatory Arrest in Thoracic Aortic Aneurysm Surgery. Texas Heart Institute Journal.

[165] Rimmer L, Fok M, Bashir M (2014). The History of Deep Hypothermic Circulatory Arrest in Thoracic Aortic Surgery. Aorta.

[166] Hughes GC, Chen EP, Browndyke JN, Szeto WY, DiMaio JM, Brinkman WT (2025). Neurocognitive Dysfunction After Short (<20 Minutes) Duration Hypothermic Circulatory Arrest. The Annals of Thoracic Surgery.

[167] Tian DH, Wan B, Bannon PG, Misfeld M, LeMaire SA, Kazui T (2013). A meta-analysis of deep hypothermic circulatory arrest versus moderate hypothermic circulatory arrest with selective antegrade cerebral perfusion. Annals of Cardiothoracic Surgery.

[168] Gega A, Rizzo JA, Johnson MH, Tranquilli M, Farkas EA, Elefteriades JA (2007). Straight deep hypothermic arrest: experience in 394 patients supports its effectiveness as a sole means of brain preservation. The Annals of Thoracic Surgery.

[169] Thudium M, Kornilov E, Hilbert T, Coburn M, Gestrich C (2021). Extended neuromonitoring in aortic arch surgery: A case series. Der Anaesthesist.

[170] Gao Z, Zhang J, Wang F, Li L, Guo Z, Wang X (2025). Near-infrared spectroscopy to monitor cerebral and renal oxygen saturation during cardiopulmonary bypass surgery for paediatric congenital heart disease: study protocol for a prospective observational cohort trial. BMJ Open.

[171] Kumar KU, Zhao Q, Bai X, Li A, Anjali P, Yu H (2015). Controlled heart rate and blood pressure reduce the life threatening aortic events and increase survival in patients with type B aortic dissection: A single center experience. International Journal of Cardiology. Heart & Vasculature.

[172] Wang Z, Ge M, Chen T, Chen C, Zong Q, Lu L (2020). Impact of hypertension on short- and long-term survival of patients who underwent emergency surgery for type A acute aortic dissection. Journal of Thoracic Disease.

[173] Nardi P, Olevano C, Bassano C, Bovio E, Cecchetti L, Forlani S (2017). The effect of postoperative malperfusion after surgical treatment of type A acute aortic dissection on early and mid-term survival. Vessel Plus.

[174] Erdoes G, Ahmed A, Kurz SD, Gerber D, Bolliger D (2023). Perioperative hemostatic management of patients with type A aortic dissection. Frontiers in Cardiovascular Medicine.

[175] Girdauskas E, Kempfert J, Kuntze T, Borger MA, Enders J, Fassl J (2010). Thromboelastometrically guided transfusion protocol during aortic surgery with circulatory arrest: a prospective, randomized trial. The Journal of Thoracic and Cardiovascular Surgery.

[176] Pupovac SS, Levine R, Giammarino AT, Scheinerman SJ, Hartman AR, Brinster DR (2022). Factor eight inhibiting bypass activity for refractory bleeding in acute type A aortic dissection repair: A propensity-matched analysis. Transfusion.

[177] El-Andari R, Bozso S, Hong Y, Moon M (2025). Anatomic and operative predictors of aortic expansion following aortic dissection repair. Scientific Reports.

[178] Fann JI, Smith JA, Miller DC, Mitchell RS, Moore KA, Grunkemeier G (1995). Surgical management of aortic dissection during a 30-year period. Circulation.

[179] Renard M, Francis C, Ghosh R, Scott AF, Witmer PD, Adès LC (2018). Clinical Validity of Genes for Heritable Thoracic Aortic Aneurysm and Dissection. Journal of the American College of Cardiology.

[180] MacGillivray TE, Gleason TG, Patel HJ, Aldea GS, Bavaria JE, Beaver TM (2022). The Society of Thoracic Surgeons/American Association for Thoracic Surgery Clinical Practice Guidelines on the Management of Type B Aortic Dissection. The Annals of Thoracic Surgery.

[181] Tsukube T (2023). Decision making and management of acute type-A dissection presenting with shock or cardiac arrest. Asian Cardiovascular & Thoracic Annals.

[182] Patel S, Desai ND, Brinster DR, Li QG, Chen EP, Sultan I (2025). Type A acute aortic dissection complicated by preoperative myocardial infarction: Insights from the International Registry of Acute Aortic Dissection. JTCVS Open.

[183] Norton EL, Longi FN, Wu X, Monaghan K, Kim KM, Fukuhara S (2024). Renal Dysfunction at Discharge and Long-Term Survival in Acute Type A Aortic Dissection. The Journal of Surgical Research.

[184] Catalano MA, Gillinov L, Lawrence KM, Toubat O, Goel NJ, Patrick WL (2025). Neighborhood socioeconomic status predicts long-term outcomes after acute type A aortic dissection repair in a propensity-matched cohort. JTCVS Open.

[185] Nappi F, Gambardella I, Singh SSA, Salsano A, Santini F, Spadaccio C (2023). Survival following acute type A aortic dissection: a multicenter study. Journal of Thoracic Disease.

[186] Matniyaz Y, Luo YX, Jiang Y, Zhang KY, Wang WZ, Pan T (2024). Short- and Long-term survival prediction in patients with acute type A aortic dissection undergoing open surgery. Journal of Cardiothoracic Surgery.

[187] Barron DJ, Livesey SA, Brown IW, Delaney DJ, Lamb RK, Monro JL (1997). Twenty-Year Follow-Up of Acute Type A Dissection: The Incidence and Extent of Distal Aortic Disease using Magnetic Resonance Imaging. Journal of Cardiac Surgery.

[188] Yu HY, Chen YS, Huang SC, Wang SS, Lin FY (2004). Late outcome of patients with aortic dissection: study of a national database. European Journal of Cardio-Thoracic Surgery.

[189] Vekstein AM, Doberne JW, Weissler EH, Wojnarski CM, Long CA, Williams AR (2023). Tailored approach and outcomes of aortic arch reconstruction after acute type A dissection repair. The Journal of Thoracic and Cardiovascular Surgery.

[190] Kreibich M, Pitts L, Kempfert J, Yildiz M, Schönhoff F, Gaisendrees C (2024). Multicentre frozen elephant trunk technique experience as redo surgery to treat residual type A aortic dissections following ascending aortic replacement. European Journal of Cardio-Thoracic Surgery.

[191] Jassar AS, Desai ND, Kobrin D, Pochettino A, Vallabhajosyula P, Milewski RK (2015). Outcomes of aortic root replacement after previous aortic root replacement: the “true” redo root. The Annals of Thoracic Surgery.

[192] An KR, de Mestral C, Tam DY, Qiu F, Ouzounian M, Lindsay TF (2021). Surveillance Imaging Following Acute Type A Aortic Dissection. Journal of the American College of Cardiology.

